# Redox regulation of m^6^A methyltransferase METTL3 in β-cells controls the innate immune response in Type 1 Diabetes

**DOI:** 10.1038/s41556-024-01368-0

**Published:** 2024-02-26

**Authors:** Dario F. De Jesus, Zijie Zhang, Natalie K. Brown, Xiaolu Li, Ling Xiao, Jiang Hu, Matthew J. Gaffrey, Garrett Fogarty, Sevim Kahraman, Jiangbo Wei, Giorgio Basile, Tariq M. Rana, Clayton Mathews, Alvin C. Powers, Audrey V. Parent, Mark A. Atkinson, Sirano Dhe-Paganon, Decio L. Eizirik, Wei-Jun Qian, Chuan He, Rohit N. Kulkarni

**Affiliations:** 1. Section of Islet Cell and Regenerative Biology, Joslin Diabetes Center; Beth Israel Deaconess Medical Center; Harvard Medical School, Boston, USA; 2. Department of Chemistry, Department of Biochemistry and Molecular Biology, and Institute for Biophysical Dynamics, The University of Chicago, Chicago, USA; 3. Howard Hughes Medical Institute, The University of Chicago, Chicago, USA; 4. Biological Sciences Division, Pacific Northwest National Laboratory, Richland, USA; 5. Present address: Department of Chemistry and Department of Biological Sciences, National University of Singapore, Singapore; 6. Institute for Genomic Medicine, University of California, San Diego, La Jolla, USA; 7. Department of Pathology, The University of Florida College of Medicine, Gainesville, USA; 8. Department of Medicine, and Department of Molecular Physiology and Biophysics, Vanderbilt University Medical Center, Nashville, USA; 9. Diabetes Center, Department of Medicine, University of California, San Francisco, San Francisco, USA; 10. Department of Biological Chemistry, and Molecular Pharmacology, Harvard Medical School, Boston, USA; 11. ULB Center for Diabetes Research, Medical Faculty, Université Libre de Bruxelles (ULB), Brussels, Belgium.

## Abstract

Type 1 Diabetes (T1D) is characterized by the destruction of pancreatic β-cells. Several observations have renewed the interest in β-cell RNA sensors and editors. Here, we report that N^6^-methyladenosine (m^6^A) is an adaptive β-cell safeguard mechanism that controls the amplitude and duration of the antiviral innate immune response at T1D onset. m^6^A writer methyltransferase 3 (METTL3) levels increase drastically in β-cells at T1D onset but rapidly decline with disease progression. m^6^A-sequencing revealed the m^6^A hypermethylation of several key innate immune mediators, including *OAS1*, *OAS2*, *OAS3,* and *ADAR1* in human islets and EndoC-βH1 cells at T1D onset. METTL3 silencing enhanced OAS levels by increasing its mRNA stability. Consistently, *in vivo* gene therapy to prolong Mettl3 overexpression specifically in β-cells delayed diabetes progression in the non-obese diabetic (NOD) mouse model of T1D. Mechanistically, the accumulation of reactive oxygen species blocked upregulation of METTL3 in response to cytokines, while physiological levels of nitric oxide enhanced METTL3 levels and activity. Furthermore, we report that the cysteines in position C276 and C326 in the zinc finger domains of the METTL3 protein are sensitive to S-nitrosylation (SNO) and are significant to the METTL3-mediated regulation of OAS mRNA stability in human β-cells. Collectively, we report that m^6^A regulates the innate immune response at the β-cell level during the onset of T1D in humans.

## MAIN TEXT

The ability to distinguish self from non-self DNA or RNA is a fundamental function of the innate immune system^1^. Consequently, several autoimmune diseases are triggered by the over-activation of the innate immune system^2^. Furthermore, significant evidence points to the activation of multiple genes that mediate the innate immune system prior to the onset of Type 1 Diabetes (T1D)^3, 4^. This gains significance since nucleic acid sensors involved in the innate immune response are upregulated in insulitic islets at T1D onset^5, 6^. Among the upregulated genes include the oligoadenylate synthase (OAS) family, a class of nucleotidyltransferases that, once activated, either act independently or produce 2ʹ–5ʹ-linked oligoadenylates to activate RNase L^7^. Notably, polymorphisms in the OAS gene cluster have been associated with susceptibility to T1D^8–10^. Intriguingly, β-cells are unique among pancreatic islet cells in possessing an ability to upregulate OAS expression in response to interferon-α or poly(I:C) (a dsRNA mimetic)^11, 12^. OAS overexpression in β-cells leads to proliferation arrest and apoptosis^12, 13^, while mice deficient in RNase L are protected from diabetes in a dsRNA-induced mouse model of T1D, consistent with the notion that over-activation of the OAS – RNase L pathway leads to β-cell death and T1D^14^.

N6-methyladenosine (m^6^A) is the most abundant modification in mRNA^15–17^. Adenosine methylation levels are regulated by “writer” proteins such as methyltransferase 3 (METTL3) and 14 (METTL14) ^15^. Several RNA binding proteins – “readers,” including YT521-B homology family proteins (e.g., YTHDF), recognize methylated adenosines and regulate several aspects of mRNA biology including mRNA decay^18–20^. METTL3 is the only enzyme in the m^6^A writer complex that presents catalytic activity ^21^. Recent work has demonstrated that METTL3 activity can be regulated by SUMOylation ^22^ and phosphorylation ^23^. However, the role of cysteine oxidative modifications such as S-nitrosylation (SNO) has not been explored.

Recent discoveries have led to the suggestion that m^6^A machinery regulates the innate immune response by accelerating the turnover of type I interferon genes in fibroblasts ^24^, via promoting adenosine-to-inosine (A-to-I) RNA editing through regulation of ADAR1^25^ or by blocking the synthesis of endogenous aberrant dsRNAs^26, 27^. However, the biological roles of m^6^A in T1D and more specifically, their contribution towards mediating β-cell innate immune responses, are virtually unknown.

Here, we show that METTL3 levels are increased at the onset of T1D followed by a rapid decline. Furthermore, we identified m^6^A hypermethylation of OAS genes and demonstrated that METTL3 downregulation in both human pseudoislets and EndoC-βH1 cells leads to the upregulation of OAS proteins. We also observed that m^6^A accelerates the mRNA decay of OAS via S-nitrosylation of the cysteine residues (C276 and C326) in the redox-sensitive zinc finger domains of METTL3. The ability of a sustained over-expression of Mettl3 in β-cells to limit the up-regulation of Oas and protect the non-obese diabetic (NOD) mouse model of T1D^28^ from developing diabetes supports the translational significance of these findings.

Together, our studies identify m^6^A as an adaptive β-cell safeguard mechanism that controls the innate antiviral immune response at the onset of T1D.

## RESULTS

### m^6^A writer (METTL3) levels peak at the onset and decrease drastically with progression of T1D

**Extended Data Fig. 1:**
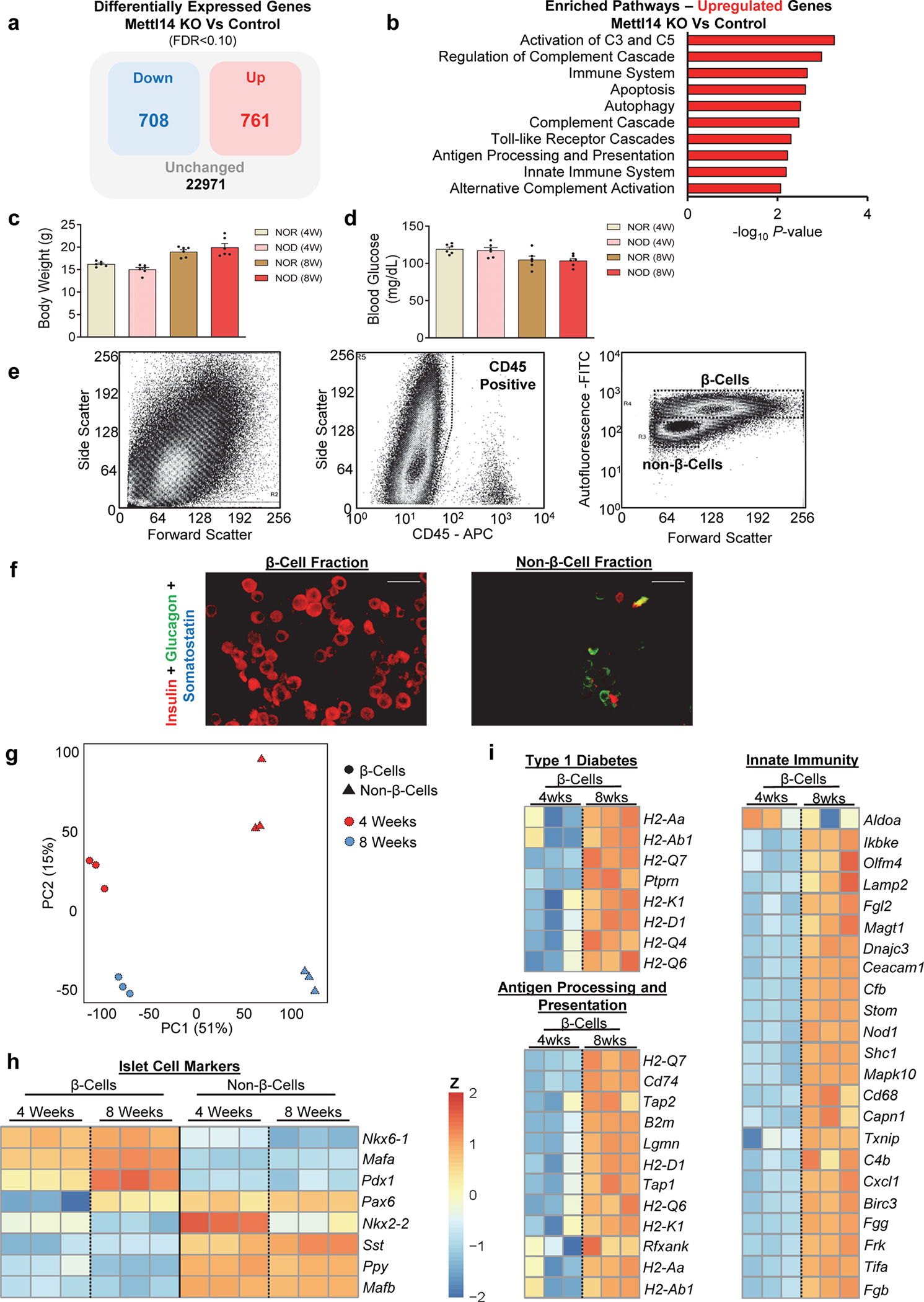
T1D pathways are upregulated in Mettl14 deficient mouse β-cells and pre-diabetic NOD β-cells show enrichment in pathways associated with Type 1 diabetes (related to Fig. 1) **a,** Diagram of differentially expressed genes in Mettl14 KO β-cells compared to controls. **b,** Pathway enrichment analyses of genes upregulated in Mettl14 KO β-cells compared to controls (Controls, n=4 pools, 2 animals/pool; M14KO, n=4 pools, 4 animals/pool). **c**, Body weight of NOR and NOD females at 4 or 8-weeks of age (n=6/group). **d**, Random-fed serum glucose levels of NOR and NOD females at 4 or 8-weeks of age (n=6/group). **e**, Representative FACS sorting strategy to deplete CD45 positive cells, and obtain an enriched β-cell population based on size, granularity, and autofluorescence (FITC) from NOD female mice. **f**, Immunofluorescence staining of sorted β- and non-β-cell fractions showing insulin staining in red, glucagon in green, and somatostatin in blue (n=3 independent experiments). Scale bar=20μM. **g**, PCA plot of RNA-seq samples of 4- or 8-week-old FACS sorted β-cells or non-β-cells (n=3 pools/group of 3 mice/pool). **h**, Heat-map representation of islet identity genes, showing enrichment for β-cell identity genes in the β-cell fraction compared to non-β-cells. **i**, Heat-map of represented genes associated with Type 1 diabetes, innate immunity, and antigen processing and presentation and upregulated in pre-diabetic 8-week-old β-cells compared to 4-week-old. All samples in each panel are biologically independent. Data were expressed as means ± SEM. Heat maps represent clipped Z-scored log CPM. Statistical analyses were performed using the Benjamini-Hochberg procedure and genes were filtered for FDR<0.05 or 0.10 in “a”. *P*-values of pathway enrichment analysis were calculated according to the hypergeometric test based on the number of physical entities present in both the predefined set and user-specified list of physical entities. Data in “a” and “b” were downloaded and reanalyzed from dataset GSE132306^35^.

We have previously reported that a decrease in m^6^A levels leads to the downregulation of genes essential for β-cell function, identity, and survival^29^. Upon further analyses of the upregulated genes (Extended Data Fig.1a), we were intrigued to find a significant enrichment in pathways related to the immune response (Extended Data Fig.1b). This interesting observation provided us with the rationale to determine if the m^*6*^A modulators, and in particular the major m^6^A writers, were impacted in T1D.

To begin, we isolated islets from NOD and an NOD congenic insulitis and diabetes-resistant model (NOR)^30^. We focused on mice that were 4- or 8 weeks of age, since at 4 weeks there is an initial phase of myeloid cell infiltration and a surge in the type I interferon signature (innate immune response), while the 8-week age marks the intense infiltration of major leukocyte subsets and T cell activation (adaptive immune response) in the NOD mouse model^31^.

**Fig. 1:**
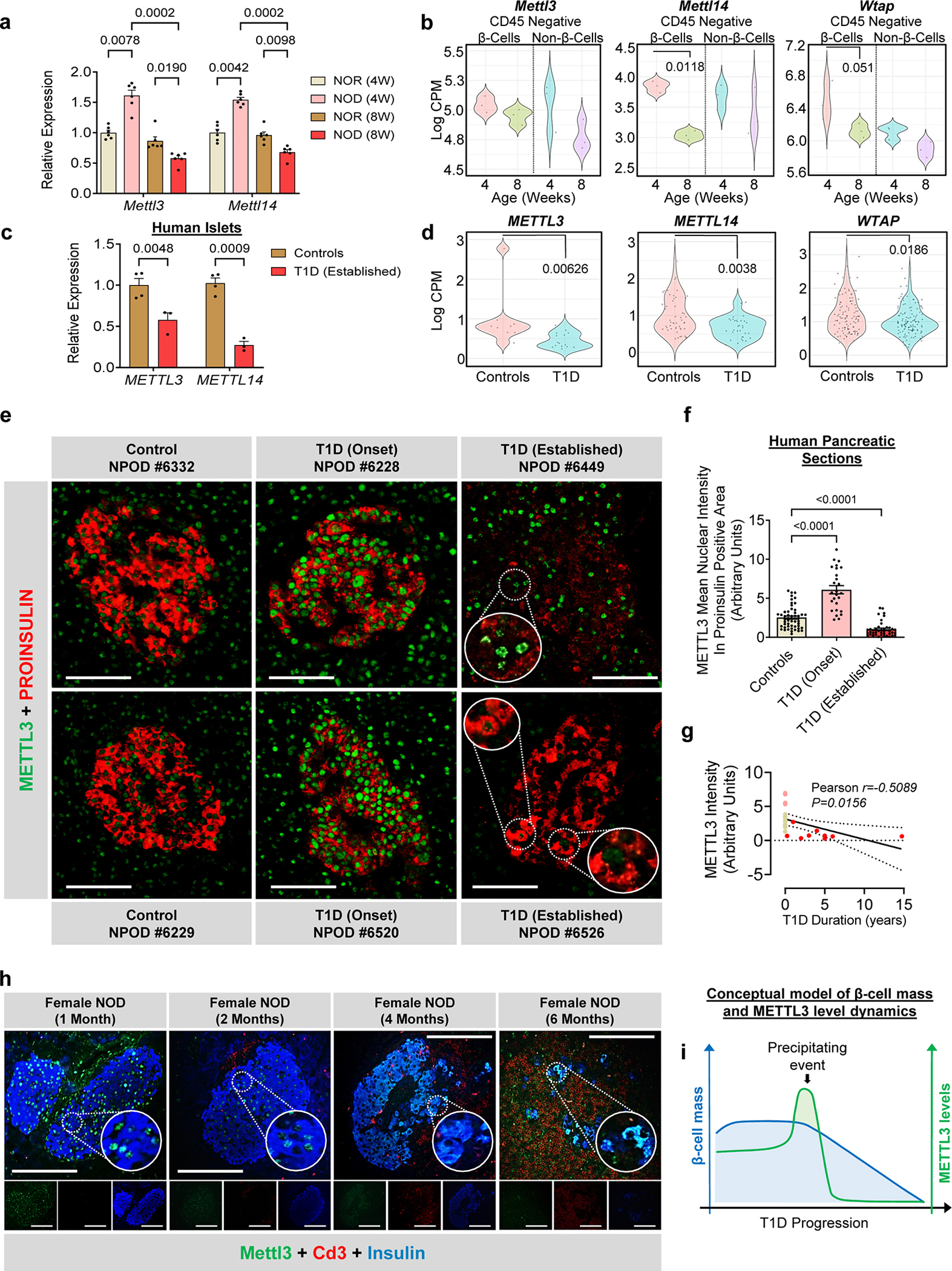
m^6^A writer METTL3 levels peak at Type 1 diabetes onset but decrease drastically with disease progression. **a,** qRT-PCR analyses of the m^6^A writer genes in whole islets isolated from 4-week-old or 8-week-old NOR or NOD mice (n=6 mice/group). **b,** Violin-plots representation of the distribution of gene expression of m^6^A writers in CD45 negative sorted β- and non-β-cells in 4- or 8-week-old NOD mice (n=3 pools of 3 mice/group). **c,** qRT-PCR analyses of *METTL3* and *METTL14* in whole islets isolated from human non-diabetic Controls (n=4), and human patients with established T1D (n=3 samples). **d,** Violin-plots representation of the distribution of gene expression of m^6^A writers in single β-cells (high insulin gene expression) from Control (n=12 human patients) and established T1D (n=4 human patients) (GSE121863). **e,** Representative pictures of immunofluorescence staining of Proinsulin + METTL3 in pancreatic sections collected from non-diabetic (Control) (n=9), T1D Onset (n=4), and established T1D (n=7) humans (scale bar = 100 μm; insert = 3x magnification; insert in NPOD #6526 =1.7x top and 3x bottom). **f,** Quantification of METTL3 intensity in proinsulin positive area in pancreatic sections collected from non-diabetic (Control) (n=52 histological fields from 9 patients), T1D Onset (n=27 histological fields from 4 patients), and established T1D (n=41 histological fields from 7 patients) humans. **g**, Pearson correlation of METTL3 nuclear intensity levels with T1D duration (Control, green; T1D onset, pink; T1D established, red). **h,** Representative pictures of immunofluorescence staining of METTL3 in pancreatic sections collected from NOD female mice with 1-, 2-, 4, and 6 months of age (n=3 mice/group) (scale bar = 50μm; insert = 3x magnification). **i,** Conceptual schematic representation of METTL3 and β-cell mass dynamics in the progression of humanT1D. All samples in each panel are biologically independent. Data were expressed as means ± SEM. Statistical analysis was performed by Two-Way ANOVA with Tukey multiple comparison test in a. Benjamini-Hochberg procedure in b and d. Two-Way ANOVA with Holm-Šídák's multiple comparisons test in c, and f. Numerical source data are available in source data.

Body weights and glucose levels did not differ between groups (Extended Data Fig.1c,d). *Mettl3* and *Mettl14* were upregulated in islets from NOD compared to NOR mice at 4 weeks of age (Fig.1a), followed by their downregulation at age 8 weeks (Fig.1a). To confirm these changes, we subjected sorted β- and non-β-cells ^32^ that were negatively selected for CD45 (Extended Data Fig.1e) to RNA-seq. The CD45-negative β-cell and non-β-cell populations were enriched for insulin and glucagon respectively (Extended Data Fig.1f) and segregated by group (Extended Data Fig.1g), with the β-cell fractions enriched for β-cell identity genes (Extended Data Fig. 1h). Consistently, β-cells from 8-week old NOD mice presented upregulation of several immune genes involved in T1D (Extended Data Fig.1j). Consistently, *Mettl14* and *Wtap* were downregulated specifically in β-cell fractions in the 8-week versus 4-week old, as well as a numerical decrease in *Mettl3* (Fig.1b).

**Extended Data Fig. 2:**
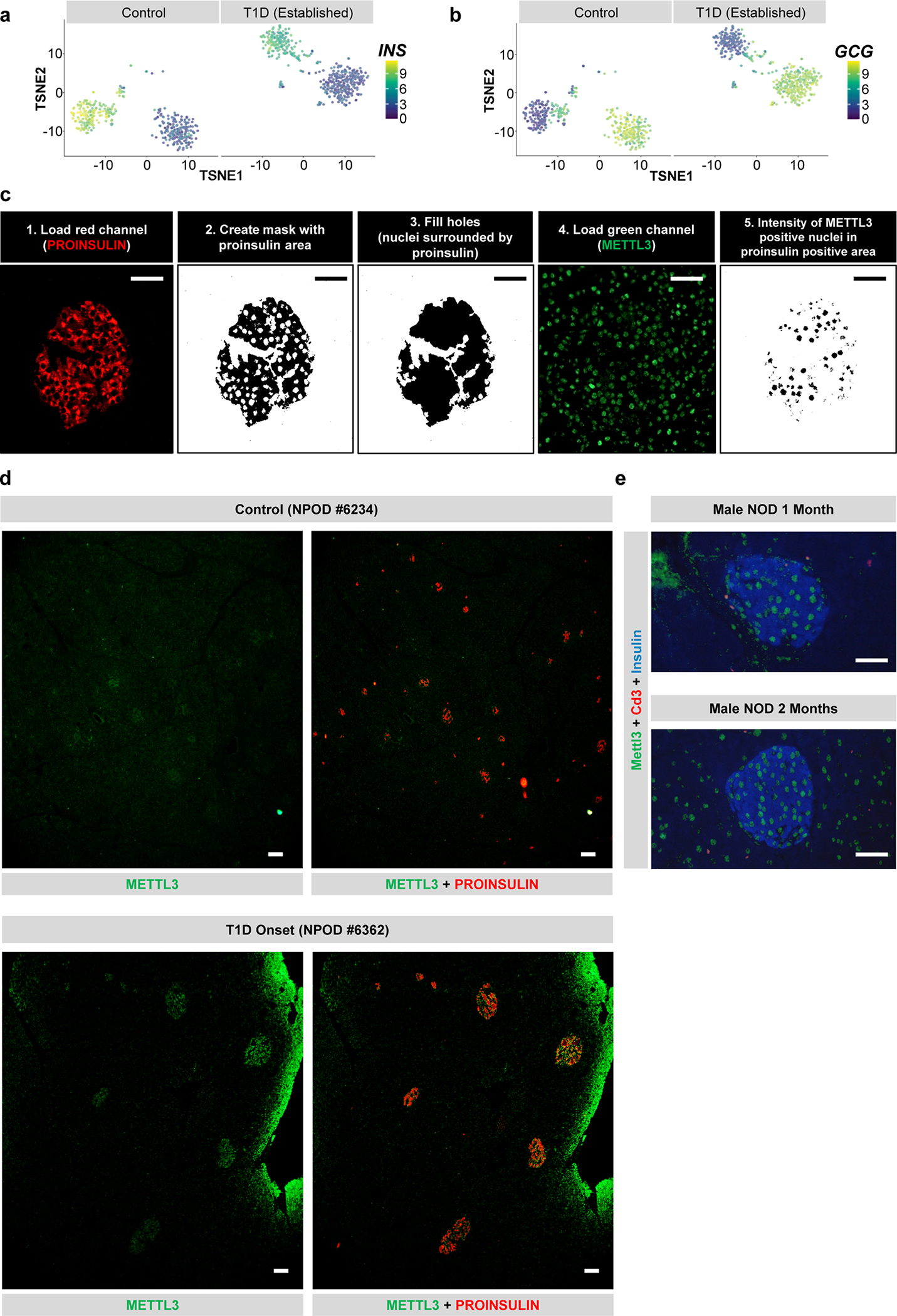
scRNA-seq re-analyses of established T1D islets, upregulation of METTL3 in human β-cells at T1D onset, and Mettl3 downregulation in β-cells with T1D progression in female NOD mice (related to Fig. 1) **a-b,** t-SNE representation of β-cells (a) (high insulin expression) or α-cells (b) (high glucagon expression) in control and established Type 1 diabetes (T1D) (GSE121863)^40^. **c,** Schematic representation of the Image J pipeline used to quantify the METTL3 nuclear intensity in proinsulin positive area in human pancreatic sections from the Network for Pancreatic Organ Donors with Diabetes (NPOD). **d,** Representative low magnification pictures of immunofluorescence staining of METTL3 (green) and Proinsulin (red) in pancreatic sections from human control and T1D onset showing a robust islet enriched upregulation of METTL3 at T1D onset. (Control, n=9; T1D Onset, n=4; established T1D, n=7). **e,** Representative pictures of immunofluorescence staining of Mettl3 (green), Insulin (blue), and Cd3 (red) in pancreatic sections from NOD male mice without any histological pancreatic immune cell infiltration patterns (n=3). Scale bar=100μM.

Next, we obtained freshly isolated islets from patients with established T1D (patient information in Supplementary Table 1). *METTL3* and *METTL14* were downregulated in established T1D islets (Fig. 1c). To confirm this, we downloaded and re-analyzed a scRNA-seq dataset performed in islets of established T1D patients and non-diabetic controls^33^. Pancreatic β-cells were identified by insulin (*INS*) gene expression (Extended Data Fig. 2a) and easily segregated from other islet cell types including α-cells (Extended Data Fig. 2b). The expression of the m^6^A writers *METTL3*, *METTL14,* and *WTAP* were downregulated in β-cells from patients with established T1D (Fig. 1d).

METTL3 is considered an attractive therapeutic target as it is the only subunit of the m^6^A writer complex possessing enzymatic activity^21, 34^. We took advantage of the abundance of METTL3 in human β-cells^29^ to perform immunofluorescence staining in pancreas sections (nPOD patient information in Supplementary Table 1). Using a pipeline on ImageJ^35^ to measure unbiased METTL3 nuclear intensity in the proinsulin + area (Extended Data Fig. 2c), we detected an upregulation of METTL3 at T1D onset, followed by downregulation in established T1D (Fig.1e,f). This downregulation correlated with the disease duration (Fig 1g). METTL3 upregulation was also noticeable in the peri-islet area at T1D onset (Fig.1e). The ease of identification of human islets from T1D onset patients even without proinsulin co-staining reflected the robust upregulation of METTL3 predominantly in islets and specifically in β-cells (Extended Data Fig. 2d).

Next, to further dissect the METTL3 dynamics in T1D progression, we performed immunostaining for Mettl3, insulin, and Cd3 (a T cell marker) in pancreas sections from NOD mice that were extensively phenotyped previously^36^ (Fig. 1h and Extended Data Fig. 2e). Consistent with human data, protein levels of Mettl3 were low in female NODs at late stages of T1D (Fig.1h), while being unaltered in age-matched males (Extended Data Fig. 2e).

Altogether T1D onset is characterized by a significant upregulation of METTL3 levels in β-cells followed by its downregulation that spans established T1D (Fig.1i) and coincides with the notion of a “precipitating event” as postulated in the Eisenbarth model of T1D^37^.

### Stimulation of human β-cells with IL-1β and IFN-α recapitulates the upregulation of METTL3

Next, we aimed to create an *in vitro* tool to recapitulate and study the METTL3 upregulation seen at T1D onset in human β-cells. Cytokine treatment of human islets, and in particular β-cells, has been reported to recapitulate several pathophysiological aspects of the molecular landscape of T1D^38, 39^.

**Fig. 2:**
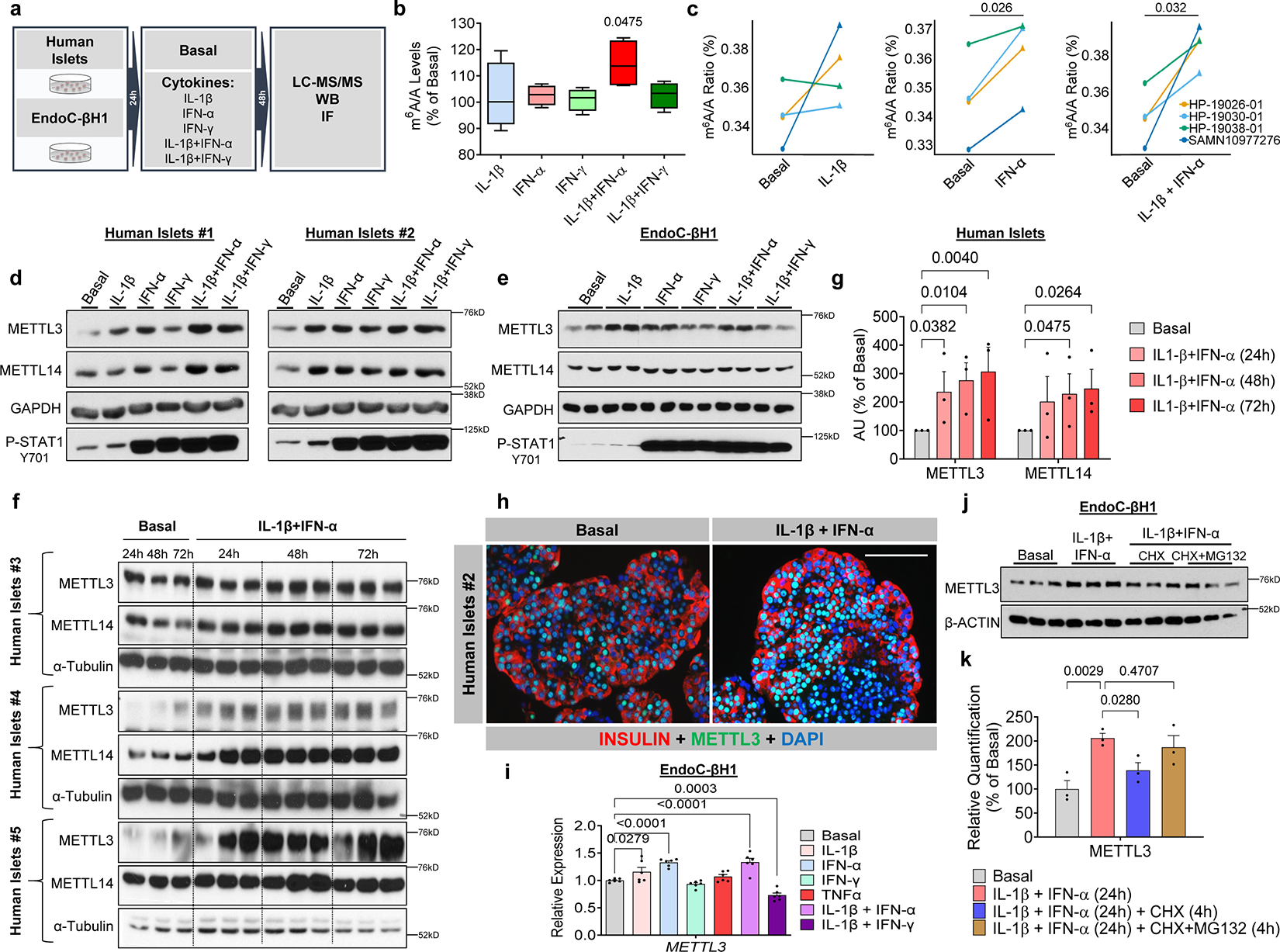
Co-treatment of human β-cells with Interleukin 1 beta (IL-1β) and interferon alpha (IFN-α) recapitulates the METTL3 upregulation seen at human T1D onset. **a,** Summary scheme of the experimental plan. **b,** m^6^A/A levels measured by LC-MS/MS in human islets treated with IL-1β, IFN-α, interferon-gamma (IFN-γ), a combination of IL-1β plus IFN-α, or IL1-β plus IFN-γ for 48h compared to PBS-treated (n=4 independent biological samples). **c,** m^6^A levels measured by LC-MS/MS in individual human islet donors treated with IL1-β, IFN-α, or IL-1β plus IFN-α compared to basal (PBS-treated) (n=4 independent biological samples). Box plot shows the median, box edges show first and third quartiles, and whiskers show the minimum and maximum. **d,** Western-blot analyses of indicated proteins in human islets treated with the represented cytokines or cytokine combinations for 48h (n=2 independent biological samples). **e,** Western-blot analyses of indicated proteins in EndoC-βH1 cells treated with the represented cytokines or cytokine combinations for 48h (n=2 independent biological samples). **f,** Western-blot analyses of indicated proteins in human islets treated with IL-1β plus IFN-α or PBS (Basal) for 24-, 48-, or 72h (n=3 independent biological samples). **g,** Protein quantification of (f). **h,** Representative pictures of immunofluorescence staining analyses of METTL3 in agar-embedded islets collected from human islets treated with IL-1β plus IFN-α or PBS (Basal) for 48h (n=2 independent biological samples) (Scale bar = 100 μm). **i**, RT-PCR analyses of *METTL3* proteins in EndoC-βH1 cells treated with the represented cytokines or cytokine combinations for 48h (n=6 independent experiments/group). **j,** Western-blot analyses of indicated proteins in EndoC-βH1 cells treated with PBS and DMSO (basal) for 24h, IL-1β + IFN-α for 24h, Il-1β + IFN-α for 24h and 10 μM cyclohexamide (CHX) for 4h, or Il-1β + IFN-α for 24h and 10μM cyclohexamide + 5μM MG132 for 4h (n=3 independent experiments/group). **k,** Protein quantification of (j). All samples in each panel are biologically independent. Data were expressed as means ± SEM. Statistical analysis was performed by One-Way ANOVA with Holm-Šídák's multiple comparisons test in b. Two-tailed paired t-test in c. Two-Way ANOVA with Fisher’s LSD test in g, i, and k. Source numerical data and unprocessed gels are available in source data.

First, we challenged human islets or a human β-cell line (EndoC-βH1)^40^ with IL-1β, IFN-α, IFN-γ, or various combinations for 48h (Fig. 2a). We then measured total m^6^A levels in mRNA by LC-MS/MS and analyzed METTL3 and METTL14 protein levels (Fig. 2a). m^6^A levels were upregulated by IFN-α and even more robustly by the combination of IL-1β + IFN-α (Fig. 2b,c). Overall, METTL3 and METTL14 protein levels increased significantly in human islets exposed to cytokines (Fig. 2d). Similar results were observed in EndoC-βH1 cells (Fig. 2e).

Next, we explored METTL3 upregulation dynamics (Fig. 2f). METTL3 and METTL14 showed a time-dependent increase in response to stimulation with IL-1β and IFN-α (Fig. 2g). We visually confirmed METTL3 upregulation upon IL-1β and IFN-α stimulation by immunostaining (Fig. 2h). Finally, to study the mechanism(s) underlying METTL3 upregulation. we first examined the transcriptional regulation of *METTL3* in EndoC-βH1 cells (Fig. 2i). *METTL3* expression did not increase more than 30% upon IL-1β, IFN-α, or IL-1β+IFN-α compared to basal (Fig. 2i). Second, to gain further insight into the stability of the METTL3 protein, we performed chase experiments (Fig. 2i and j). Interestingly, METTL3 upregulation was blocked with cyclohexamide, and this was rescued with MG132 (Fig. 2i and j). These findings suggest that the upregulation of METTL3 is primarily mediated by increased protein synthesis concomitantly with a modest transcriptional upregulation. Overall, these results demonstrate that the stimulation of human islets and β-cells with IL-1β and IFN-α *in vitro* accurately reproduces the upregulation of METTL3 observed during the onset of human T1D.

### m^6^A landscape analyses reveal hypermethylation of antiviral innate immune genes with cytokines

**Fig. 3:**
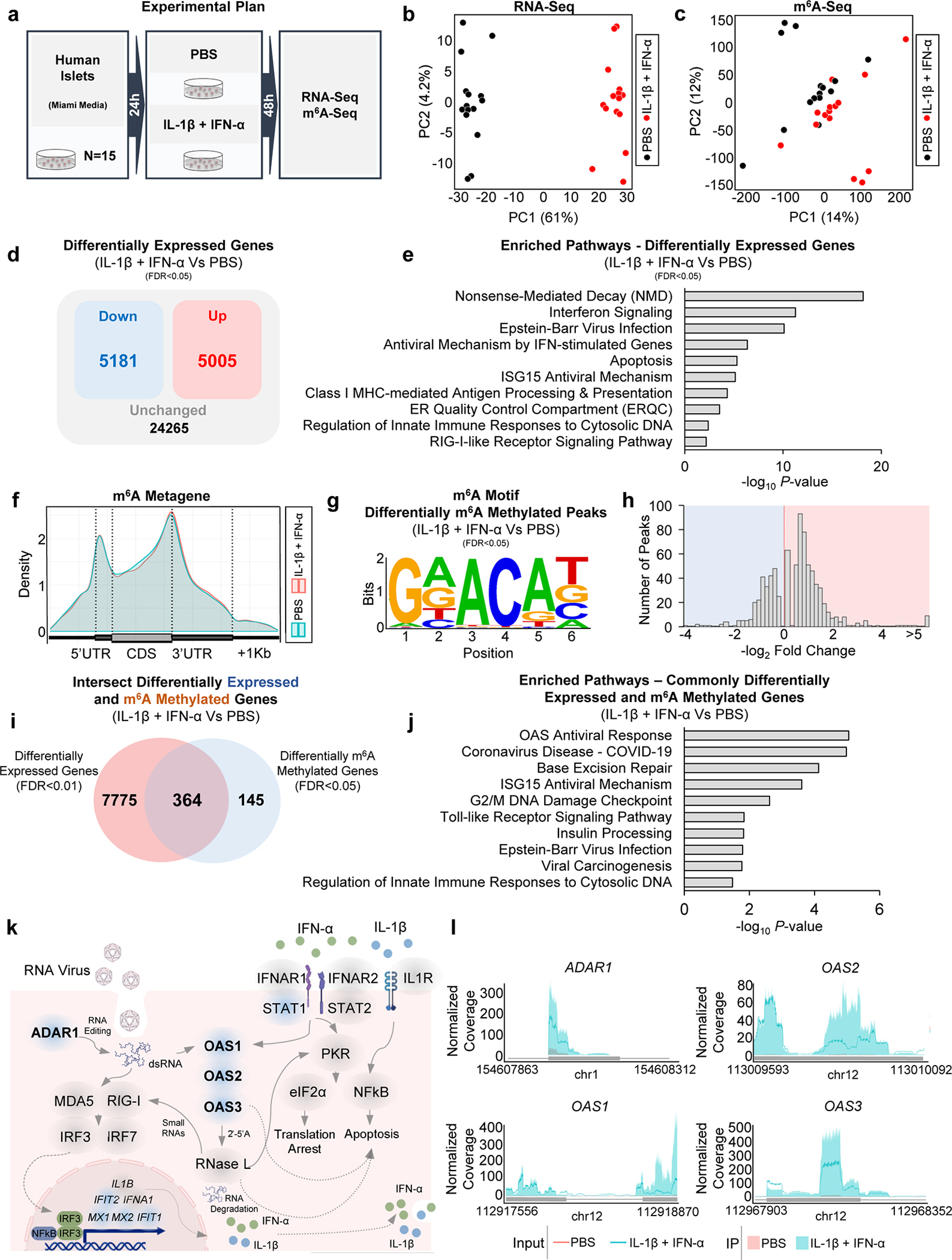
m^6^A landscape analyses of human islets treated with IL-1β and IFN-α reveal hypermethylation of 2′,5′-oligoadenylate synthetase (OAS) genes. **a,** Summary scheme of the experimental plan. **b,** PCA plot of RNA-sequencing in human islets treated with PBS (black dots) or IL-1β and IFN-α (red dots) (n=15 human patients). **c,** PCA plot of m^6^A-sequencing in human islets treated with PBS (black dots) or IL-1β and IFN-α (red dots) (n=15 human patients). **d,** Diagram representation of the upregulated (red), downregulated (blue), and unchanged genes (black) in human islets treated with IL-1β and IFN-α compared to PBS. Statistical analyses were performed using the Benjamini-Hochberg procedure and genes were filtered for FDR<0.05. **e,** Pathway enrichment analyses of upregulated and downregulated genes in human islets treated with IL-1β and IFN-α compared to PBS. **f,** Metagene of m^6^A enriched peaks in PBS- (blue) and IL-1β plus IFN-α-treated (red) human islets. **g,** Enrichment for known m^6^A consensus motif RRACH. **h,** Histogram of log_2_-fold change showing the distribution of differential m^6^A loci fold changes from IL-1β plus IFN-α-treated versus PBS. **i,** Venn diagram representation of the intersection between differentially methylated and expressed genes in human islets treated with IL-1β and IFN-α compared to PBS (n=15 human patients). Statistical analyses were performed using the Benjamini-Hochberg procedure and differentially expressed genes were filtered for FDR<0.01 and m^6^A methylated genes for FDR<0.05. **j,** Pathway enrichment analyses of intersected genes in (i). **k,** Representation of antiviral innate immune pathway based on KEEG and Wikipathway annotations depicting several m^6^A hypermethylated genes (blue shade) and unchanged genes (grey shade) in human islets treated with IL-1β and IFN-α compared to PBS-treated (genes filtered for FDR<0.05). **l,** Coverage plots of m^6^A peaks in *ADAR1*, *OAS1*, OAS2, and *OAS3* genes in human Islets treated with L-1β and IFN-α (blue) or PBS (red). Plotted coverages are the median of the n replicates presented. All samples in each panel are biologically independent. *P*-values of pathway enrichment analysis were calculated according to the hypergeometric test based on the number of physical entities present in both the predefined set and user-specified list of physical entities. Source numerical data are available in source data.

To directly evaluate the m^6^A landscape during T1D onset, we employed RNA-seq and m^6^A-seq of islets from 15 human donors (Supplementary Table 1) stimulated with cytokines (IL-1β+IFN-α) for 48h (Fig. 3a). The transcriptome (Fig. 3b) and m^6^A methylome (Fig. 3c) of cytokine-treated islets showed segregation from PBS-treated samples. However, the m^6^A response was heterogeneous among donors (Fig. 3c). Transcriptomic changes induced by cytokines were characterized by the downregulation of 5181 genes and upregulation of 5005 genes (Fig. 3d). Analyses of the combined differentially regulated gene sets revealed enrichment in pathways involved in nonsense-mediated decay and innate immune pathways (Fig. 3e).

Next, analyses of the m^6^A methylome showed changes that were consistent with patterns from previous studies^16, 17^ in that the m^6^A peaks were enriched at the start and stop codons (Fig. 3f), and characterized by the canonical GGACU motif (Fig. 3g). m^6^A-sequencing revealed 800 differently methylated sites in 509 genes with a higher number of hypermethylated m^6^A sites in cytokine-treated compared to PBS-treated islets (Fig. 3h). Since IL-1β and IFN-α stimulation of human islets led to extensive transcriptomic remodeling, we hypothesized that some of these pathways are modulated by m^6^A at the mRNA level.

**Extended Data Fig. 3:**
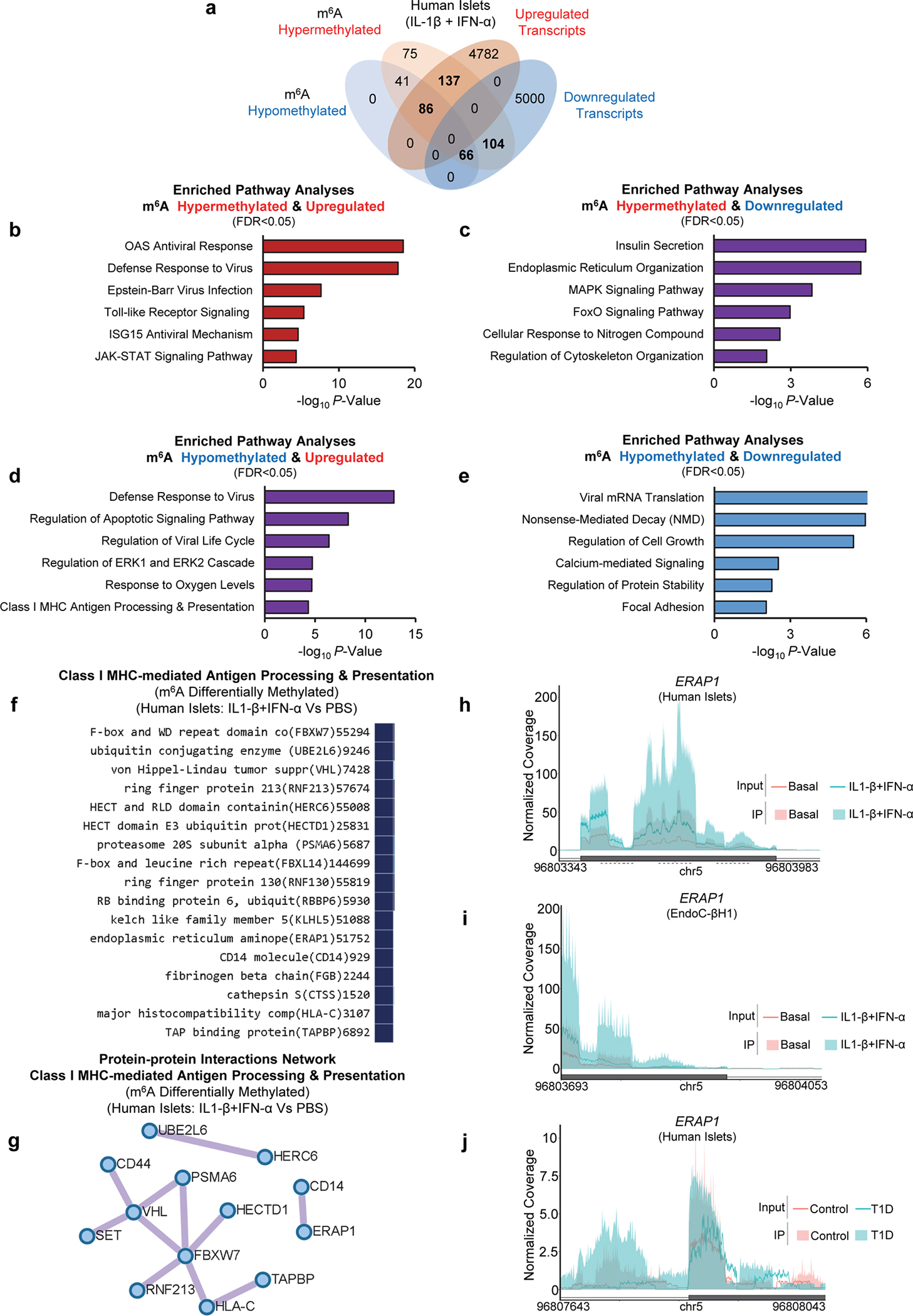
m^6^A landscape analyses of human islets treated with IL-1β and IFN-α reveal differential methylation of class I MHC-mediated antigen processing and presentation genes (related to Fig. 3) **a,** Venn diagram representation of the m^6^A hypermethylated, m^6^A hypomethylated, upregulated, or downregulated genes in human islets treated with IL-1β and IFN-α compared to PBS. Statistical analyses were performed using the Benjamini-Hochberg procedure and genes were filtered for FDR<0.05. **b-e**, Pathway enrichment analyses of m^6^A hypermethylated and upregulated (a), m^6^A hypermethylated and downregulated (c), m^6^A hypomethylated and upregulated (d), or m^6^A hypomethylated and downregulated genes (e) in human islets treated with IL-1β and IFN-α compared to PBS. *P*-values were calculated according to the hypergeometric test based on the number of physical entities present in both the predefined set and user-specified list of physical entities. **f**, MHC class I differentially m^6^A methylated genes in human islets treated with IL-1β and IFN-α compared to PBS. **g**, Protein-protein interactions network of class I MHC-mediated antigen processing and presentation differentially m^6^A methylated in human islets treated with IL-1β and IFN-α compared to PBS. **h-j**, Coverage plots of m^6^A peaks *ERAP1*in human islets treated with IL-1β and IFN-α or PBS (h) (n=15/group), EndoC-βH1 cells treated with IL-1β and IFN-α or PBS (i) (n=6/group), or human T1D and Control islets (j) (Controls, n=20; T1D, n=7). Plotted coverages are the median of the n replicates presented. All samples in each panel are biologically independent. *P*-values of pathway enrichment analysis were calculated according to the hypergeometric test based on the number of physical entities present in both the predefined set and user-specified list of physical entities.

To test this hypothesis, we intersected the differentially expressed (DEGs) and m^6^A methylated genes (DMGs) (Figure 3i and Extended Data Fig. 3a). Pathway enrichment analyses on these common 364 intersected genes (Fig. 3i) revealed interconnected pathways involving the antiviral innate immune system (Fig. 3j). Specifically, several double-strand RNA sensors such as OAS and the RNA editor *ADAR1*, were highly hypermethylated and upregulated in human islets in response to IL-1β and IFN-α (Fig. 3k, I and Extended Data Fig. 3b). Other hypermethylated and upregulated genes included several interferon-induced genes such as interferon-induced protein with tetratricopeptide (*IFIT1* and *IFIT2*), and MX dynamin-like GTPase (*MX1* and *MX2*) (Fig. 3k). Conversely, genes displaying hypermethylation and subsequent downregulation exerted influences on pathways linked to insulin secretion (Extended Data Fig. 3c). Additionally, a set of hypomethylated genes exhibited enrichment in antiviral defense pathways (Extended Data Fig. 3d,e), including multiple MHC class I genes responsible for antigen presentation (Extended Data Fig. 3f,g), such as endoplasmic reticulum aminopeptidase 1 (*ERAP1*) (Extended Data Fig. 3h,I,j). This enzyme is known to play a critical role in trimming peptides for antigen presentation^41^, including the preproinsulin signal peptide antigen^42^. Overall, these results indicate a dynamic transcriptomic remodeling in human islets upon IL-1β and IFN-α stimulation and show that genes that are differentially expressed and m^6^A-decorated are mainly involved in the antiviral innate immune response.

### OAS genes are upregulated and m^6^A decorated in human β-cells treated with IL-1β and IFN-α

**Extended Data Fig. 4:**
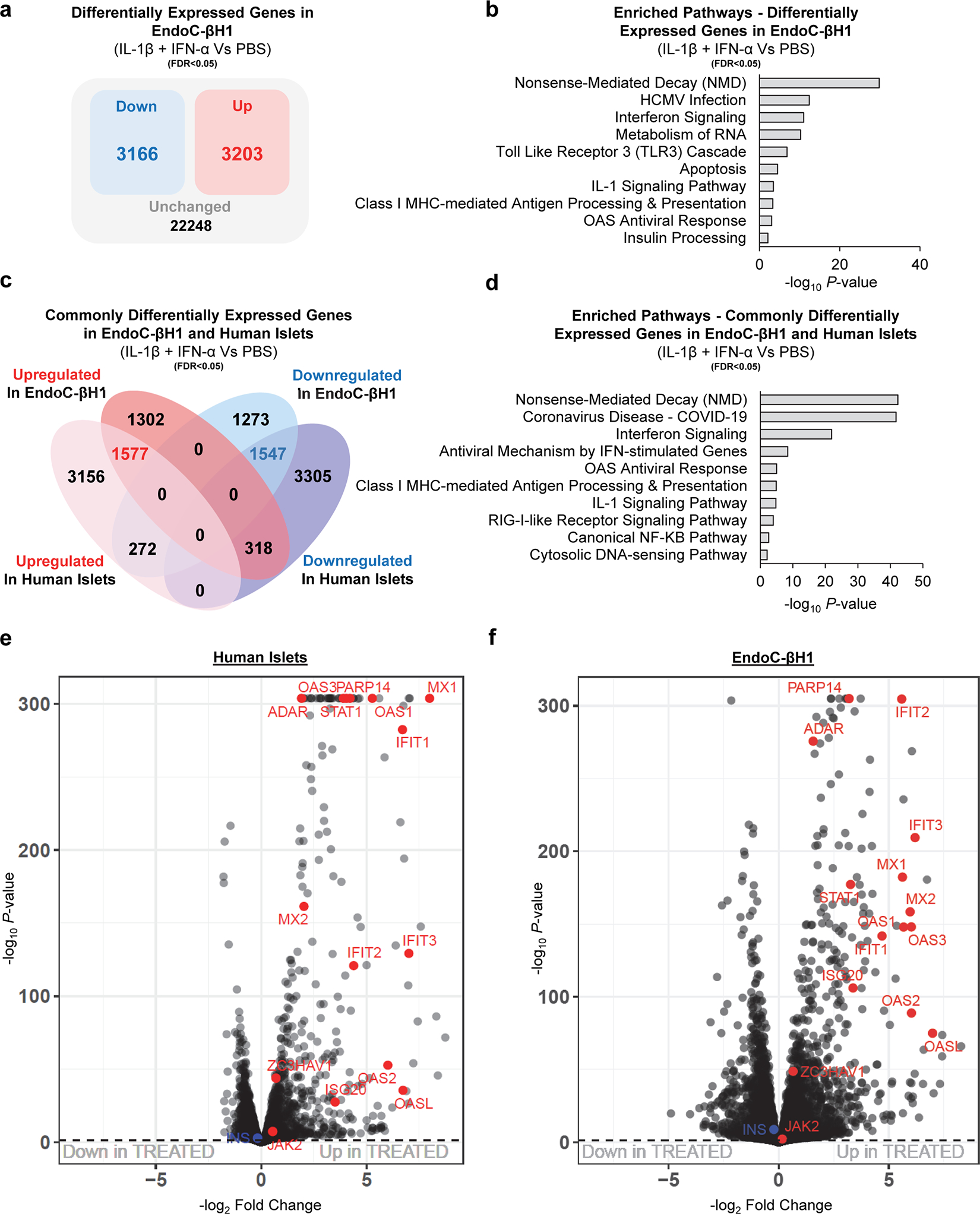
Human islets and EndoC-βH1 cells present an extensive overlap in the innate immune response to IL-1β and IFN-α (related to Fig. 3 and 4) **a,** Diagram representation of the upregulated (red), downregulated (blue), and unchanged genes (black) in EndoC-βH1 cells treated with IL-1β and IFN-α compared to PBS. Statistical analyses were performed using the Benjamini-Hochberg procedure and genes were filtered for FDR<0.05. **b,** Pathway enrichment analyses of upregulated and downregulated genes in EndoC-βH1 cells treated with IL-1β and IFN-α compared to PBS. **c,** Venn diagram representation of the commonly upregulated (red), downregulated (blue), and unchanged genes (black) of the intersected genes in EndoC-βH1 and human islets cells treated with IL-1β and IFN-α compared to PBS. Statistical analyses were performed using the Benjamini-Hochberg procedure and genes were filtered for FDR<0.05. **d,** Pathway enrichment analyses of commonly upregulated and downregulated genes in human islets and EndoC-βH1 cells treated with IL-1β and IFN-α compared to PBS. **e-f,** Volcano-plot representation of differentially expressed genes in human islets (e) and EndoC-βH1 cells (f) treated with IL-1β and IFN-α compared to PBS. Innate immune genes are depicted in red and show a near absolute overlap between human islets and EndoC-βH1 cells. Human islets: n=15 biologically independent samples. EndoC-βH1 cells: n=6 biologically independent samples. Statistical analyses were performed using the Benjamini-Hochberg procedure. *P*-values of pathway enrichment analysis were calculated according to the hypergeometric test based on the number of physical entities present in both the predefined set and user-specified list of physical entities.

**Fig. 4:**
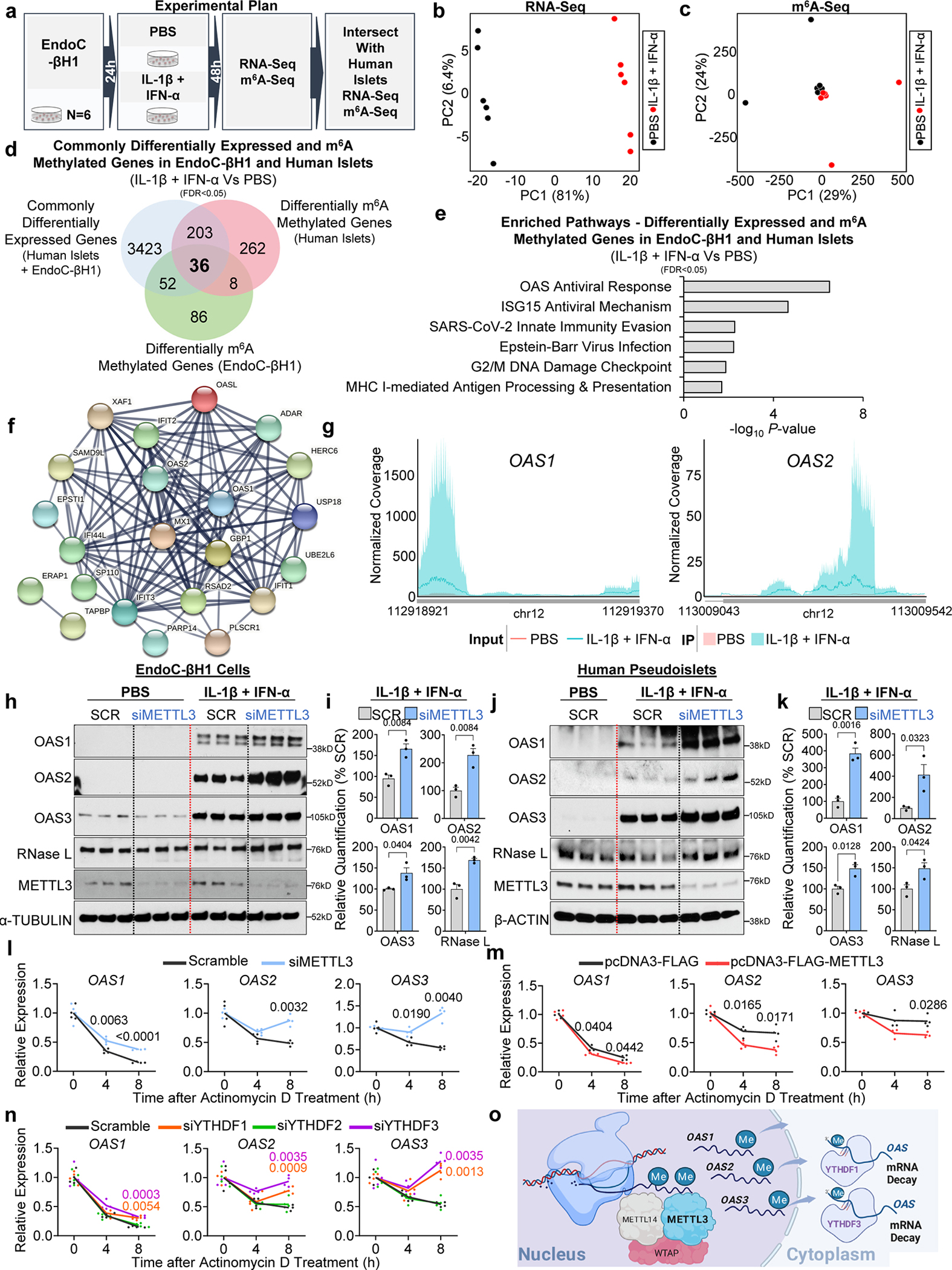
m^6^A controls the mRNA stability of 2′-5′-oligoadenylate synthetase (OAS) genes. **a,** Summary scheme of experimental plan. **b,** PCA plot of RNA-sequencing in EndoC-βH1 cells treated with PBS (black dots) or IL-1β and IFN-α (red dots) (n=6 independent experiments/group). **c,** PCA plot of m^6^A-sequencing in EndoC-βH1 treated with PBS (black dots) or IL-1β and IFN-α (red dots) (n=6 independent experiments/group). **d,** Venn diagram representation of the intersection between differentially expressed genes in human islets and EndoC-βH1 treated with IL-1β and IFN-α compared to PBS-treated with differentially m^6^A methylated genes in human islets and EndoC-βH1 treated with IL-1β and IFN-α compared to PBS-treated. Statistical analyses were performed using the Benjamini-Hochberg procedure and genes were filtered for FDR<0.05. **e,** Pathway enrichment analyses of intersected genes in (d). **f,** STRING functional protein-protein interaction network of 36 intersected genes, showing differential expression and m^6^A methylation in human islets and Endoc-βH1 cells treated with IL-1β and IFN-α compared to PBS. **g**, Coverage plots of m^6^A peaks in *OAS1* and *OAS2* genes in EndoC-βH1 cells treated with L-1β and IFN-α or PBS. Plotted coverages are the median of the n replicates presented. **h,** Western-blot analyses of indicated proteins after IL-1β plus IFN-α or PBS stimulation in EndoC-βH1 cells harboring METTL3 KD or scramble (SCR) (n=3 independent experiments/group). Same experiment of Extended Data Fig 5h, with same loading control. **i,** Protein quantification of indicated protein in (h). **j,** Western-blot analyses of indicated proteins after IL-1β plus IFN-α or PBS stimulation in human pseudoislets METTL3 KD or SCR (n=3 biological independent samples). **k,** Protein quantification of indicated protein in (j). **l** qRT-PCR analyses of OAS genes after IL-1β plus IFN-α stimulation of METTL3 KD or scramble EndoC-βH1 cells after a time-course treatment with actinomycin D (ActD) (n=4 independent experiments/group). **m,** qRT-PCR analyses of OAS genes after IL-1β plus IFN-α stimulation in METTL3 overexpressing (OE) and control (FLAG) EndoC-βH1 cells after a time-course treatment with ActD (n=4 independent experiments/group). **n,** qRT-PCR analyses of OAS genes after IL-1β plus IFN-α stimulation in scramble, YTHDF1, YTHDF2, or YTHDF3 KD EndoC-βH1 cells after a time-course treatment with ActD (n=4 independent experiments/group). **o,** Model depicting the role of METTL3-mediated m^6^A hypermethylation of OAS genes in response to IL-1β and IFN-α or at T1D onset that leads to OAS nuclear export to the β-cell cytoplasm and recognition by m^6^A readers YTHDF1 and YTHDF3 leading to accelerated mRNA decay. All samples in each panel are biologically independent. Data were expressed as means ± SEM. Statistical analysis was performed by two-tailed unpaired t-test in i and k, Two-Way ANOVA with Holm-Šídák's multiple comparisons test in l, m, and n. Or as otherwise stated above. *P*-values of pathway enrichment analysis were calculated according to the hypergeometric test based on the number of physical entities present in both the predefined set and user-specified list of physical entities. Source unprocessed gel images and numerical data are available in source data.

To validate the m^6^A regulation of OAS genes specifically in human β-cells, we challenged EndoC-βH1 cells with cytokines (IL-1β+IFN-α) or PBS (Fig. 4a). The transcriptome (Fig. 4b) and the m^6^A methylome (Fig. 4c) of cytokine-treated cells showed clear segregation between groups. Cytokine stimulation resulted in the downregulation of 3166 genes and the upregulation of 3203 genes compared to PBS-treated EndoC-βH1 cells (Extended Data Fig. 4a). Pathway enrichment analyses of the combined upregulated and downregulated genes identified nonsense-mediated decay and antiviral innate immune pathways (Extended Data Fig. 4b).

Since human islets are composed of several cell types, we focused on the differentially expressed genes in human islets that overlapped with EndoC-βH1 cells to ensure β-cell specificity. Intersection of the differentially expressed genes in human islets and EndoC-βH1 cells revealed 1577 and 1547 commonly upregulated and downregulated genes respectively (Extended Data Fig. 4c). Enrichment pathway analyses on the intersected genes, again revealed nonsense mediated decay and the antiviral innate immune response (Extended Data Fig. 4d). The gene expression similarities between human islets (Extended Data Fig 4e) and EndoC-βH1 cells (Extended Data Fig. 4f) in response to cytokines was remarkable. Indeed, virtually all innate immune sensor genes were consistently upregulated in both models (Extended Data Fig. 4e,f). The translational relevance of these findings is supported by the observation that numerous innate immune genes that were commonly upregulated in both human islets and EndoC-βH1 cells treated with IL-1β and IFN-α overlap with those upregulated in the human insulitic islets from T1D onset patients, and notably include the *OAS1*, *OAS2*, and *OAS3* genes^8^.

**Extended Data Fig. 5:**
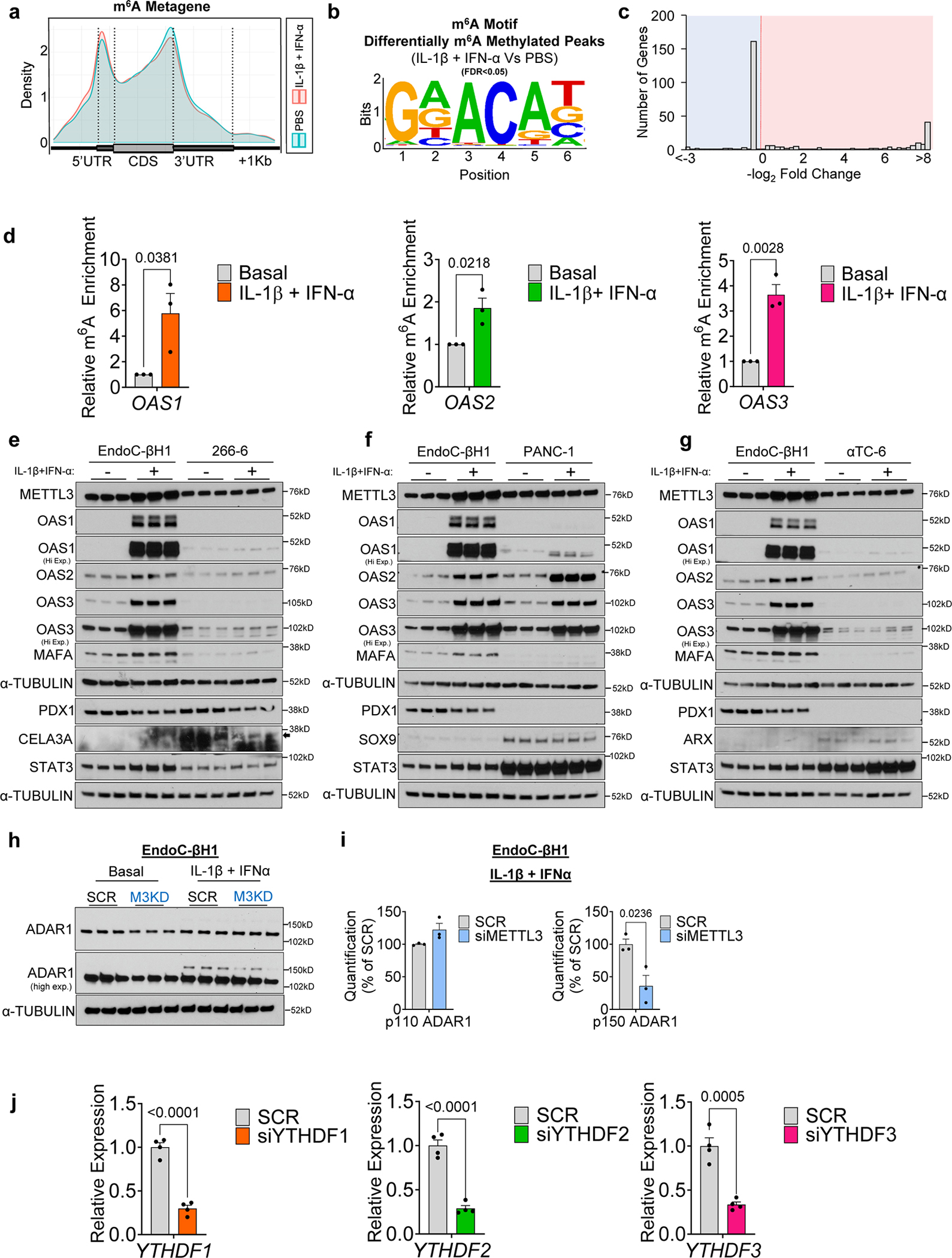
OAS upregulation is more prominent in β-cells, and METTL3 silencing leads to the downregulation of ADAR1 p150 isoform in β-cells (related to Fig. 4) **a,** Metagene of m^6^A enriched peaks in PBS (blue) or IL-1β + IFN-α-treated (red) EndoC-βH1 cells. **b,** Enrichment for known m^6^A consensus motif RRACH. **c,** Histogram of the distribution of differential m^6^A loci log_2_ fold changes from IL-1β plus IFN-α-treated versus PBS in EndoC-βH1 cells. **d**, m^6^A-IP-qPCR showing increased m^6^A in OAS1, OAS2, and OAS3 in IL-1β + IFN-α-treated EndoC-βH1 cells compared to PBS (Basal) (n=3/group). **e-g**, Western-blot analyses of indicated proteins in EndoC-βH1 cells and 266–6 cells (e), or PANC-1 (f), or αTC-6 (g) treated with PBS or IL1-β plus IFN-α for 24h (n=3 experiments). **h**, Western-blot analyses of indicated proteins EndoC-βH1 cells harboring METTL3 silencing or Scramble and treated with PBS or IL1-β plus IFN-α (n=3 experiments). Same experiment of Fig 4h, with same loading control. **i**, Protein quantification of indicated proteins related to (h). **j**, qRT-PCR analyses of YTHDF genes after IL-1β plus IFN-α stimulation in scramble, YTHDF1, YTHDF2, or YTHDF3 KD EndoC-βH1 cells (n=4/group). All samples in each panel are biologically independent. Data were expressed as means ± SEM. Statistical analysis was performed by two-tailed unpaired t-test.

Examination of the m^6^A methylome revealed m^6^A peaks enriched at the start and stop codons (Extended Data Fig. 5a) and characterized by the canonical GGACU motif (Extended Data Fig. 5b). Analysis of m^6^A-sequencing revealed 301 differently methylated sites in 182 genes and a higher number of sites with increased levels of m^6^A methylation in IL-1β and IFN-α-treated compared to PBS (Extended Data Fig. 5c).

To further explore the m^6^A regulation of the OAS innate immune sensors in β-cells, we intersected the commonly expressed and m^6^A methylated genes in human islets or EndoC-βH1 cells (Fig. 4d). This analysis identified 36 commonly expressed and m^6^A-regulated genes mainly involved in the innate immune pathway including *OAS* and *ADAR1* (Fig. 4e,f). Protein-protein interaction analyses on the common m^6^A-regulated and expressed genes revealed a close relationship among OAS1, OAS2, and OAS3 proteins with other innate immune mediators (Fig. 4f). Importantly, we were able to validate the m^6^A hypermethylation of *OAS1* and *OAS2* in EndoC-βH1 cells by m^6^A-Seq (Fig. 4g), and *OAS1*, *OAS2*, and *OAS3* by m^6^A-IP-qPCR in EndoC-βH1 cells (Extended Data Fig. 5d).

Finally, the observation that different pancreatic islet cell types are capable of mounting divergent antiviral responses^12^ prompted us to dissect the OAS response in other pancreatic cells. To this end, we challenged EndoC-βH1, 266–6 (mouse acinar cell line) (Extended Data Fig. 5e), PANC-1 (human duct cell line) (Extended Data Fig. 5f), and α-TC6 (mouse α-cell lines) (Extended Data Fig. 5g) with IL-1β and IFN-α or PBS in parallel for 48h. These data showed that METTL3 and OAS upregulation in response to cytokines was prominent in human β-cells.

### m^6^A mRNA methylation promotes OAS mRNA decay in human β-cells

Next, to test the hypothesis that m^6^A impacts the upregulation of OAS protein levels we challenged EndoC-βH1 cells harboring METTL3 knock-down (KD) or control scramble with cytokines (IL-1β + IFN-α) or PBS for 16h (Fig. 4h). OAS1, OAS2, and OAS3 were upregulated in scramble cells stimulated with cytokines compared to PBS (Fig. 4h). Furthermore, METTL3 silencing led to an even greater increase in OAS protein levels in response to cytokines (Fig. 4h,i). A similar approach in human pseudoislets (Fig. 4j) with METTL3 silencing exhibited a greater upregulation of OAS proteins than scramble controls (Fig. 4j,k). Furthermore, we noted alterations in the expression of other RNA sensors and editors (Extended Data Fig. 5h). For example, METTL3 silencing in EndoC-βH1 cells led to downregulation of isoform p150 of ADAR1 (Extended Data Fig. 5i), in contrast to the upregulation of OAS, suggesting that m^6^A regulates RNA editing in β-cells. Consistently, ADAR1 has recently been shown to be induced by cytokines and to increase the A-to-I RNA editing of Alu-Containing mRNAs in human β-cells^43^and ADAR1p150 has been reported to prevent fatal auto-inflammation^44^.

Next, we explored whether METTL3 controls the innate immune response in β-cells by accelerating the mRNA decay of OAS genes. To this end, we challenged EndoC-βH1 cells harboring either METTL3 KD or scramble KD with actinomycin D (a transcription inhibitor) or DMSO in the presence of cytokines (Fig. 4l). We observed that METTL3 silencing increases the mRNA stability of OAS genes (Fig. 4l). To validate these findings, we asked whether the converse, i.e. upregulating METTL3, accelerates decay of OAS genes. Indeed, overexpression of METTL3 in EndoC-βH1 cells accelerated the decay of OAS genes in response to cytokines (Fig. 4m).

To validate the m^6^A regulation of *OAS* expression and identify the putative m^6^A reader proteins controlling OAS mRNA decay, we independently silenced the readers, YTHDF1, YTHDF2, or YTHDF3, in EndoC-βH1 cells (Fig. 4n and Extended Data Fig. 5j). YTHDF1 or YTHDF3 downregulation increased *OAS* mRNA stability, suggesting that m^6^A hypermethylation of *OAS* transcripts accelerates the decay of their mRNAs mediated by YTHDF1 and YTHDF3 (Fig. 4n). In summary, these data reveal that METTL3 upregulation and consequent m^6^A hypermethylation of *OAS1*, *OAS2,* and *OAS3* control the innate immune response in human β-cells by promoting their mRNA decay via YTHDF1 and YTHDF3 (Fig. 4o).

### OAS upregulation leads to an extensive downregulation of metallothioneins in β-cells

**Fig. 5:**
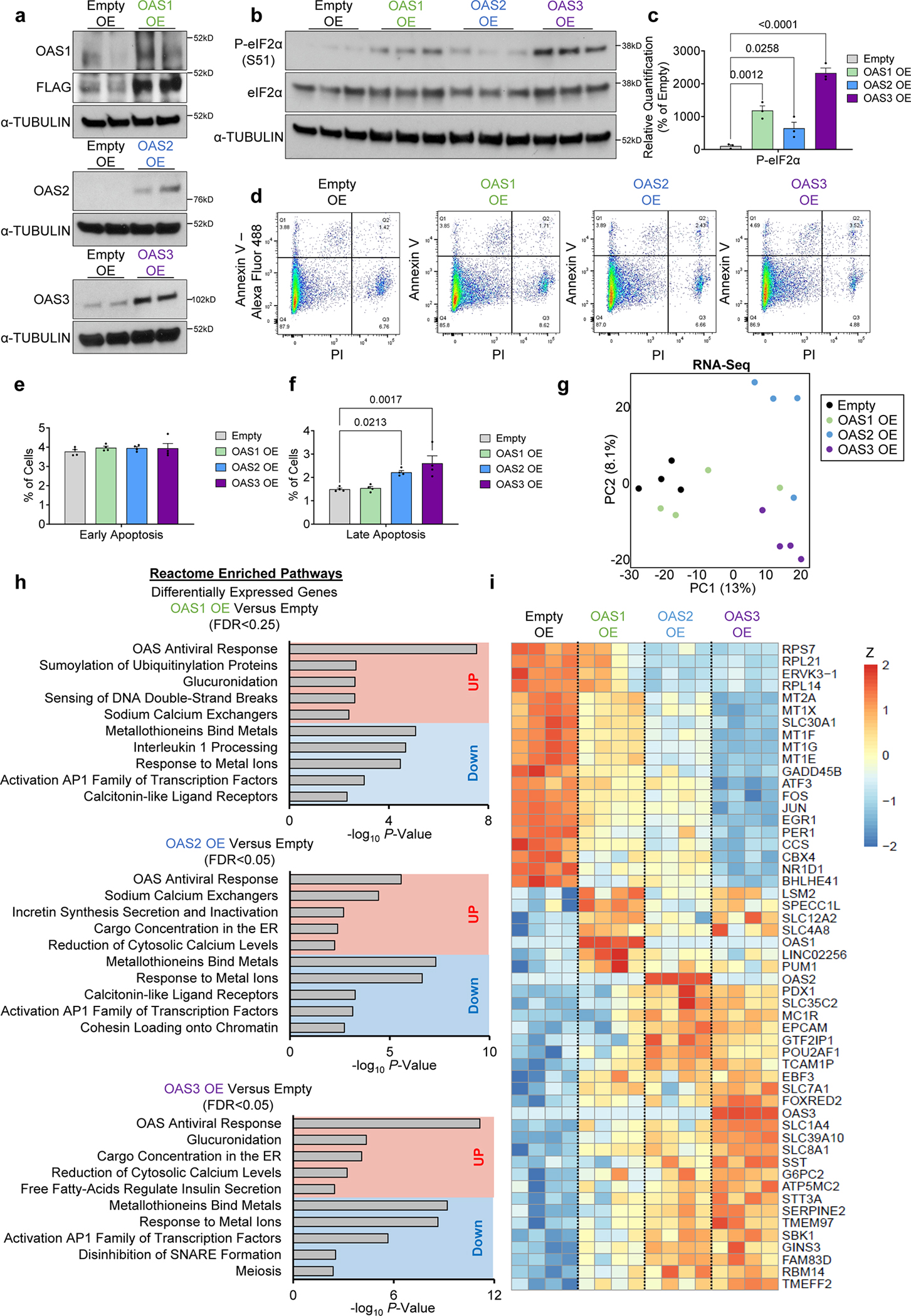
OAS upregulation leads to an extensive downregulation of metallothioneins in β-cells. **a,** Western-blot validation of OAS1, OAS2, or OAS3 protein overexpression in EndoC-βH1 cells (n=2 experiments). **b,** Western-blot analyses of indicated proteins in EndoC-βH1 cells overexpressing OAS1, OAS2, OAS3 or a stuffer control plasmid for 72h (n=3 independent experiments/group). **c,** Protein quantification of (b). **d,** Flow cytometry analyses of PI and Annexin V in EndoC-βH1 cells overexpressing OAS1, OAS2, OAS3, or a stuffer control plasmid (n=4 independent experiments/group). **e-f,** Quantification of (d), showing early (e) and late (f) apoptosis rates. **g,** PCA plot of RNA-sequencing in EndoC-βH1 cells overexpressing OAS1 (green), OAS2 (blue), OAS3 (purple) or a stuffer control (black) plasmid for 72h (n=4 independent experiments/group). **h,** Pathway enrichment analyses of differentially expressed genes. **i**, Heat-map representation of top differentially expressed genes. All samples in each panel are biologically independent. Heat map represents clipped Z-scored log CPM. Data were expressed as means ± SEM. Statistical analysis was performed by Benjamini-Hochberg procedure or One-Way ANOVA with Holm-Šídák's multiple comparisons test in c and f. *P*-values of pathway enrichment analysis were calculated according to the hypergeometric test based on the number of physical entities present in both the predefined set and user-specified list of physical entities. Source unprocessed gel images and numerical data are available in source data.

To gain insight into the transcriptomic alterations induced by OAS in human β-cells, we individually overexpressed OAS1, OAS2, or OAS3 in EndoC-βH1 cells in the basal cellular state (Fig. 5a).

Notably, the overexpression of OAS proteins induced the phosphorylation of eukaryotic initiation factor-2α (eIF2α) at serine 51, even in the absence of interferon stimulation, indicative of translation inhibition (Fig. 5b,c). Moreover, OAS2 and OAS3 overexpression, but not OAS1, resulted in elevated β-cell apoptosis rates (Fig. 5d,e,f). The impact of OAS upregulation in β-cells remains relatively unexplored and to further clarify this phenomenon, we conducted RNA-seq analyses in EndoC-βH1 cells overexpressing OAS1, OAS2, or OAS3 individually (Fig. 5g). Our analyses revealed clear distinctions, with OAS2 and OAS3 overexpression producing more profound changes in the transcriptomic profiles as compared to OAS1 (Fig. 5g). Pathway enrichment analyses using the Rotation Gene Set Test (Roast) method (see methods) showed upregulation of pathways associated with the OAS response and endoplasmic reticulum function. In stark contrast, OAS upregulation resulted in a consistent and widespread downregulation of metallothionein genes, including *MT1E*, *MT1X*, and *MT2A* (Fig. 5i). Metallothioneins (MTs) are low molecular weight, cysteine-rich proteins known for their high-affinity binding to heavy metals^45, 46^. They play pivotal roles in metal metabolism and detoxification and, owing to their numerous cysteine residues, function as potent antioxidants^45, 46^. Notably, the overexpression of MTs in β-cells has been demonstrated to confer broad resistance to oxidative stress^45^. Thus, our findings indicate that OAS overexpression triggers a widespread downregulation of MTs, compromising the antioxidant capacity of the β-cells and ultimately rendering them more susceptible to apoptosis.

### m^6^A landscape of established T1D is enriched in β-cell identity and function genes

**Fig. 6:**
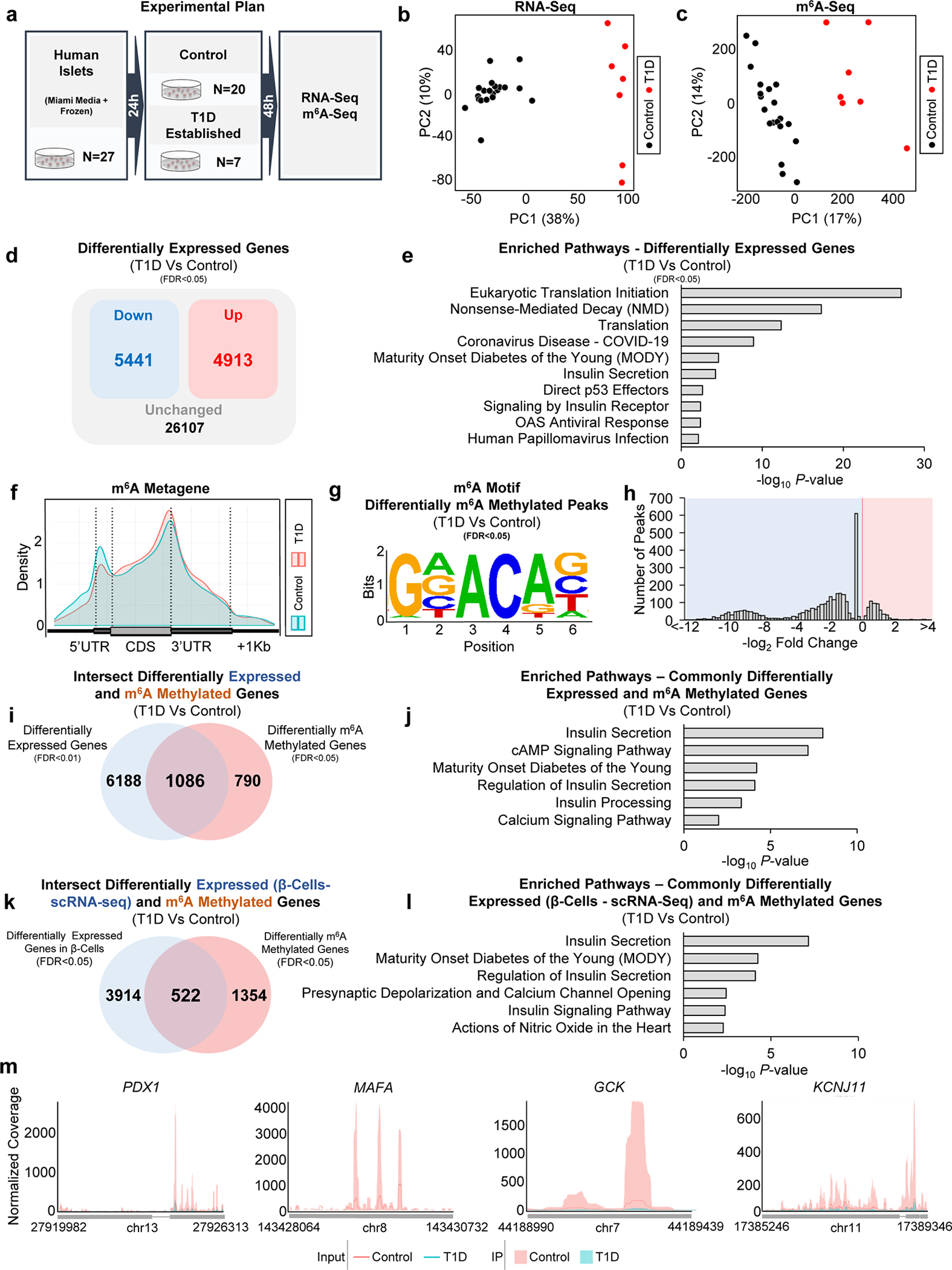
m^6^A landscape of established T1D is enriched in β-cell identity and function genes. **a,** Summary scheme of the experimental plan. **b,** PCA plot of RNA-sequencing in human islets from Control (black dots) (n=20 biological independent samples) or established T1D (red dots) (n=7 biological independent samples). **c,** PCA plot of m^6^A- in human islets from Control (black dots) (n=20 biological independent samples) or established T1D (red dots) (n=7 biological independent samples). **d,** Diagram representation of the upregulated (red), downregulated (blue), and unchanged genes (black) in human islets from Control or T1D. **e,** Pathway enrichment analyses of upregulated and downregulated genes in human islets from T1D compared to Controls. **f,** Metagene of m^6^A enriched peaks in Control (blue) and T1D (red) human islets. **g,** Enrichment for known m^6^A consensus motif RRACH. **h,** Histogram showing the distribution of differential m^6^A loci log_2_ fold changes from T1D versus Control human islets. **i,** Intersection of differentially expressed and m^6^A methylated genes in T1D compared to Control human islets. **j,** Pathway enrichment analyses of intersected genes in (i). **k,** Intersection of differentially expressed genes in T1D β-cells compared to Control from a published scRNA-seq dataset^33^ and our m^6^A dataset comparing the differentially m^6^A methylated genes in established T1D compared to Control human islets. **l,** Pathway enrichment analyses of intersected genes in (k). **m,** Coverage plots of m^6^A peaks in β-cell identity genes in human islets from established T1D compared to Controls. Plotted coverages are the median of the n replicates presented. Human islets: Controls n=20 and T1D n=7 biologically independent samples. EndoC-βH1 cells: n=6 biologically independent samples. Statistical analyses were performed using the Benjamini-Hochberg procedure. *P*-values of pathway enrichment analysis were calculated according to the hypergeometric test based on the number of physical entities present in both the predefined set and user-specified list of physical entities. Source numerical data are available in source data.

To explore the m^6^A landscape of established T1D, we performed RNA-seq and m^6^A-seq in human islets from patients with established T1D or non-diabetic controls (Fig. 6a). The transcriptome (Fig. 6b) and the m^6^A methylome (Fig. 6c) exhibited clear segregation in the principal component analysis (PCA). T1D islets presented downregulation of 5441 genes and an upregulation of 4913 genes compared to controls (Fig. 6d). Pathway enrichment analyses of the combined upregulated and downregulated genes (Fig. 6d) revealed pathways involved in translation initiation, nonsense-mediated decay, insulin secretion, and maturity-onset of diabetes of the young (MODY) (Fig. 6e).

m^6^A-seq analysis confirmed the enrichment of m^6^A peaks at the start and stop codons (Fig. 6f) and was characterized by the canonical GGACU motif (Fig. 6g). There were 3485 differently methylated sites in 1876 genes and a higher number of sites presenting m^6^A hypomethylation in established T1D compared to controls (Fig. 6h) consistent with our findings of downregulation of METTL3 with T1D progression (Fig. 1e,f).

**Extended Data Fig. 6:**
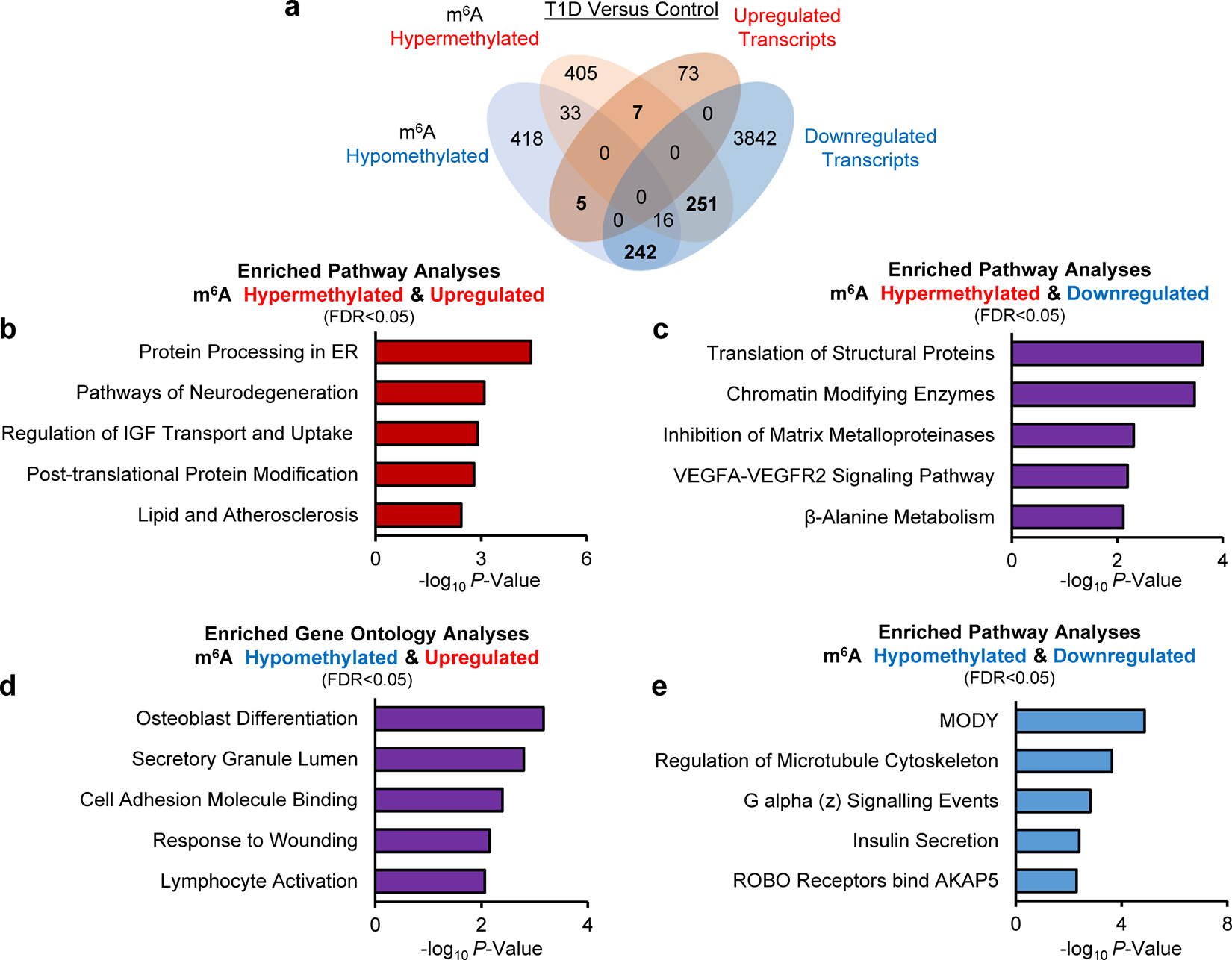
m^6^A landscape analyses of established T1D reveal differential methylation of master regulators of β-cell identity and function (related to Fig. 6) **a,** Venn diagram representation of the m^6^A hypermethylated, m^6^A hypomethylated, upregulated, or downregulated genes in human islets from patients with established T1D or non-diabetic Controls. Statistical analyses were performed using the Benjamini-Hochberg procedure and genes were filtered for FDR<0.05. Human islets: Controls n=20 and T1D n=7 biologically independent samples. **b-e**, Pathway enrichment analyses of m^6^A hypermethylated and upregulated (a), m^6^A hypermethylated and downregulated (c), m^6^A hypomethylated and upregulated (d), or m^6^A hypomethylated and downregulated genes (e) in human islets from established T1D compared to Controls. *P*-values of pathway enrichment analysis were calculated according to the hypergeometric test based on the number of physical entities present in both the predefined set and user-specified list of physical entities.

To dissect the m^6^A regulation of gene expression in established T1D, we intersected and performed pathway enrichment analysis on the differentially expressed and m^6^A methylated genes in human islets from patients with established T1D (Fig. 6i). This analysis identified enrichment for pathways associated with β-cell function and identity (Fig. 6j). Next, to overcome the limitations of the number of insulin-positive β-cells being drastically decreased in established T1D, we intersected the differentially m^6^A decorated genes with the β-cell transcriptome from a sc-RNA seq dataset performed in islets from established T1D patients and non-diabetic controls (GSE121863) (Fig. 6k and Extended Data Fig. 6a). Pathway analyses on the intersected differentially expressed and m^6^A methylated genes revealed enrichment for insulin secretion and MODY (Fig. 6l and Extended Data Fig. 6b–e). Several master regulators of β-cell function and identity, such as *PDX1*, *MAFA*, *GCK*, and *KCNJ11* presented m^6^A hypomethylation and were downregulated in established T1D (Extended Data Fig. 6e; Fig. 6m). Together, these results identify distinct m^6^A landscapes at the onset and in established T1D specifically in human β-cells and point to m^6^A regulation of β-cell function and identity genes in established disease.

### Sustained over-expression of Mettl3 in β-cells delays diabetes progression in the NOD mouse

**Extended Data Fig. 7:**
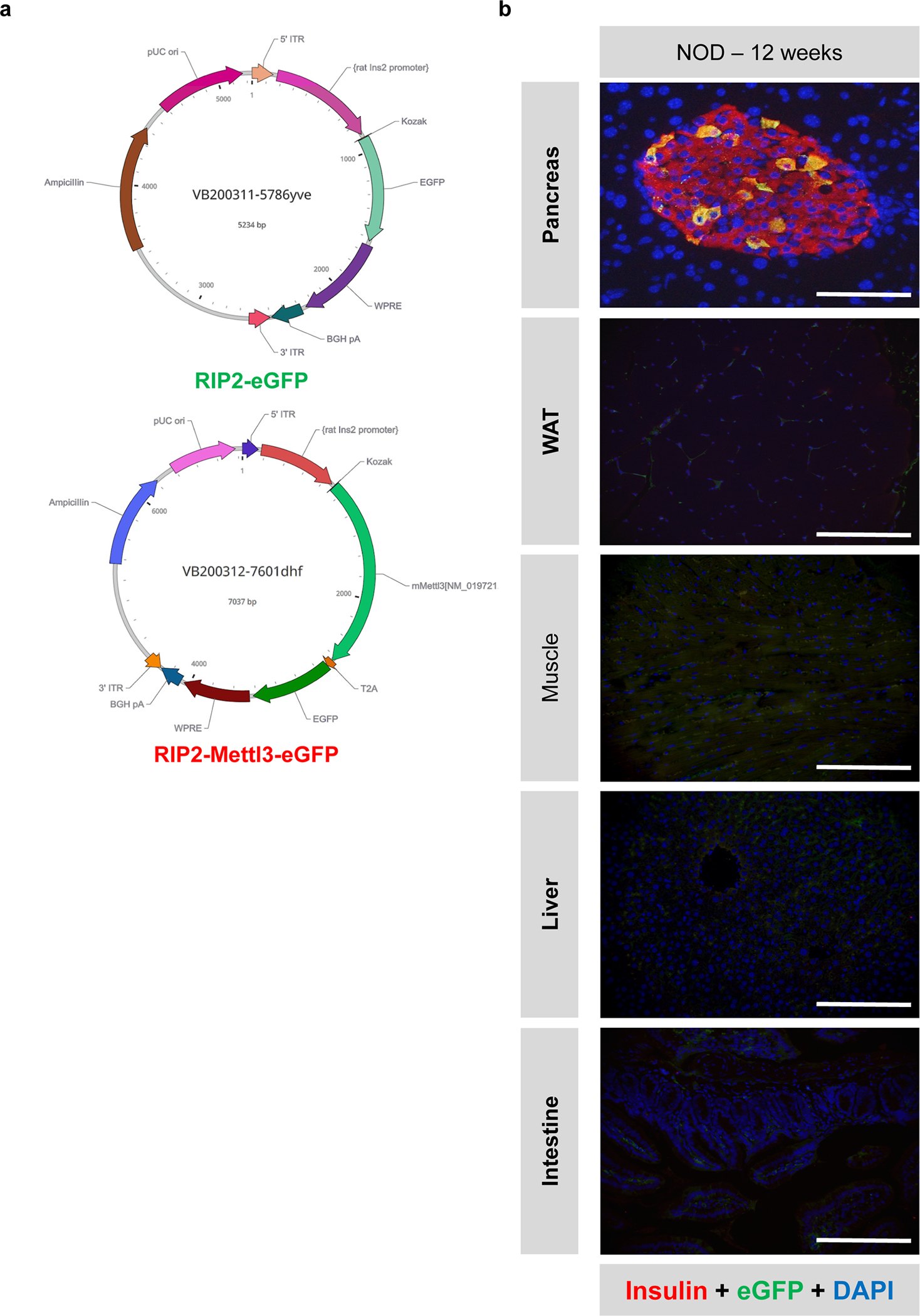
Mettl3 overexpression in NOD β-cells (related to Fig. 7) **a,** Schematic diagram showing the vector design of AAV8 driving eGFP or Mettl3 under the control of a rat insulin promoter II. **b**, Immunofluorescence images of pancreas, white adipose tissue (WAT), muscle, liver, and intestine showing insulin (red), eGFP (green), and DAPI (blue). Scale bar=100μM.

**Fig. 7:**
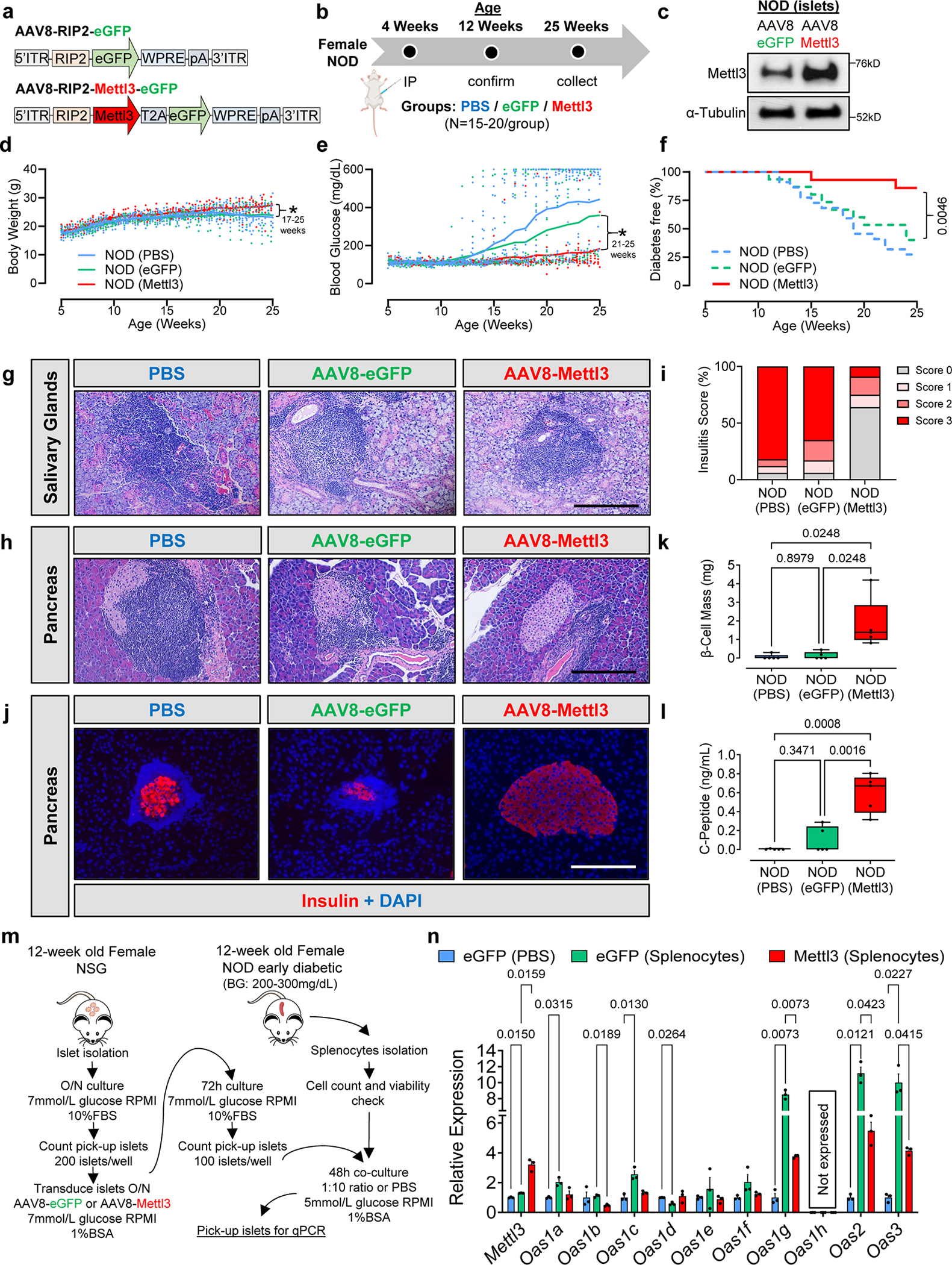
*In vivo* AAV8-mediated overexpression of Mettl3 in NOD Mouse β-Cells delays Type 1 diabetes progression. **a,** Schematic diagram showing the construction of AAV8 driving eGFP or Mettl3 under the control of a rat insulin promoter II. **b,** Scheme of experimental approach depicting NOD mice receiving PBS (blue), AAV8 overexpressing eGFP (green), or AAV8 overexpressing Mettl3 (red). N=20 mice in NOD (PBS), and n=15 mice in NOD (eGFP) or NOD (Mettl3). **c,** Western-blot validation of Mettl3 overexpression in isolated NOD female islets after 8 weeks of *in vivo* transduction (n=3 independent experiments). **d,** Body weight trajectories of NOD (PBS) (blue dots/line), NOD (eGFP) (green dots/line), and NOD (Mettl3) (red dots/line) (*P*=0.0215, 17 weeks of age; *P*=0.0034, 25 weeks of age, NOD - PBS vs. NOD - Mettl3). **e,** Blood glucose trajectories of NOD (PBS) (blue dots/line), NOD (eGFP) (green dots/line), and NOD (Mettl3) (red dots/line) (*P*=0.0239, 21 weeks of age; *P*=0.0028, 25 weeks of age, NOD - PBS vs. NOD - Mettl3). **f,** Percentage diabetes-free NOD (PBS), NOD (eGFP), and NOD (Mettl3) at the end of the 25 weeks of age. **g,** Representative hematoxylin and eosin (H&E) staining showing immune cell infiltration in salivary glands in NOD (PBS), NOD (eGFP), or NOD (Mettl3) (scale bar, 200 μm) (n=5 mice/group). **h,** Representative H&E staining showing immune cell infiltration in pancreatic islets in NOD (PBS), NOD (eGFP), or NOD (Mettl3) (scale bar, 200 μm) (n=5 mice/group). **i,** Quantification of insulitis score of pancreatic sections from H (n=5/group). **j,** Representative immunofluorescence images showing insulin (red) and DAPI (blue) in pancreatic sections from NOD (PBS), NOD (eGFP), or NOD (Mettl3) (scale bar, 200 μm). **k,** β-cell mass estimations of NOD (PBS), NOD (eGFP), and NOD (Mettl3) at 25 weeks of age (n=5 mice/group). **l,** Serum C-peptide levels in NOD (PBS), NOD (eGFP), and NOD (Mettl3) at 25 weeks of age (n=5 mice/group). **m,** Schematic representation of the co-culture experimental plan. **n,** qRT-PCR analyses of *Oas* genes in NOD *scid* gamma (NSG) islets transduced with eGFP and co-cultured with PBS (blue bars) or NOD diabetogenic splenocytes (green bars), or NSG islets transduced with AAV8 overexpressing Mettl3 and co-cultured with NOD diabetogenic splenocytes (red bars) (n=3/group; islets from 3 pools of 5 mice each pool). All samples in each panel are biologically independent. Data were expressed as means ± SEM. Statistical analysis was performed by mixed effect analysis with Dunnet’s multiple comparison test in d and Sidak multiple comparison test in e. Log-rank (Mantel-cox) test in f. Two-Way ANOVA with Holm-Sidak’s multiple comparisons test in k, l, and n. Source numerical data and unprocessed gel images are available in source data.

To test whether prolonged upregulation of Mettl3 in the NOD mouse β-cells would lead to a faster turnover and/or decrease in expression of Oas and delay T1D progression we designed two different adeno-associated virus serotype 8 (AAV8) driving eGFP or Mettl3 under the control of the rat insulin promoter II (RIP2)^47^ (Fig. 7a and Extended Data Fig. 7a). We chose to infuse PBS or AAV8 into 4-week-old animals (Fig. 7b) because our data showed that Mettl3 levels start to decline after this age in NOD females (Fig. 1a,h). We confirmed Mettl3 protein overexpression and specificity at 12 weeks of age (Fig. 7c and Extended Data Fig. 7b) and followed these mice for up to 25 weeks of age, similar to previous studies^48–50^. Body weight trajectories did not change until 17 weeks of age, when mice that received AAV8 driving eGFP overexpression (AAV8-eGFP) or PBS started to lose weight compared to mice that received AAV8 driving Mettl3 overexpression (AAV8-Mettl3) (Fig. 7d). Random-fed blood glucose levels increased with age in AAV8-eGFP and PBS groups, in contrast to the AAV8-Mettl3 group (Fig. 7e) indicating a delayed progression of T1D (Fig. 7f).

**Extended Data Fig. 8:**
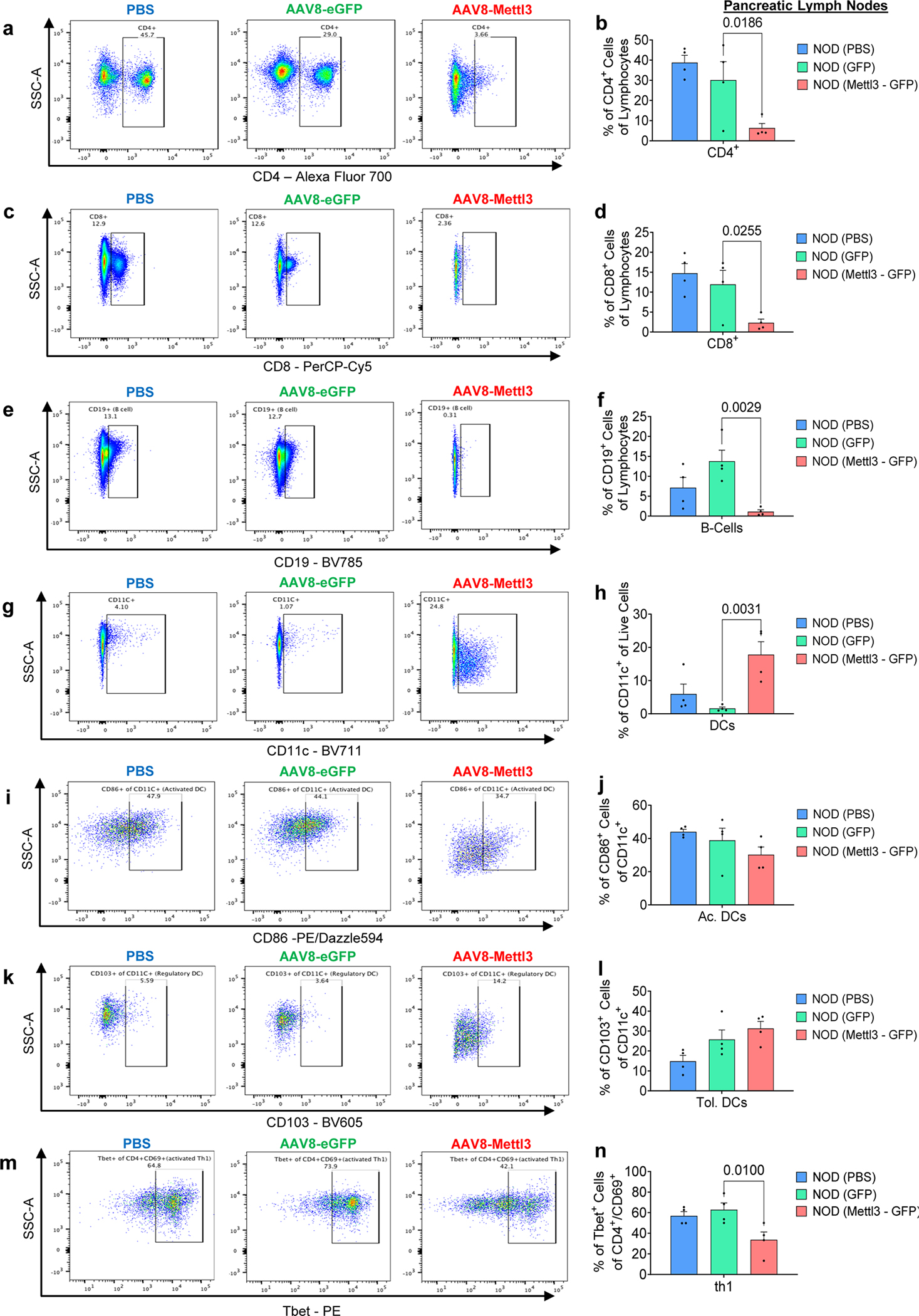
Immune cell profiling of NOD pancreatic lymph nodes (related to Fig. 7) **a-n,** Flow cytometry analyses of CD4 (a,b), CD8 (c,d), B-cells (e,f), dendritic cells (g,h), activated dendritic cells (i,j), tolerogenic dendritic cells (k,l), or th1 cells (m,n) isolated from pancreatic lymph nodes of NOD mice receiving PBS, eGFP, or Mettl3 overexpression (n=4/group). All samples in each panel are biologically independent. Data were expressed as means ± SEM. Statistical analysis was performed by One-Way ANOVA with Holm-Sidak test.

**Extended Data Fig. 9:**
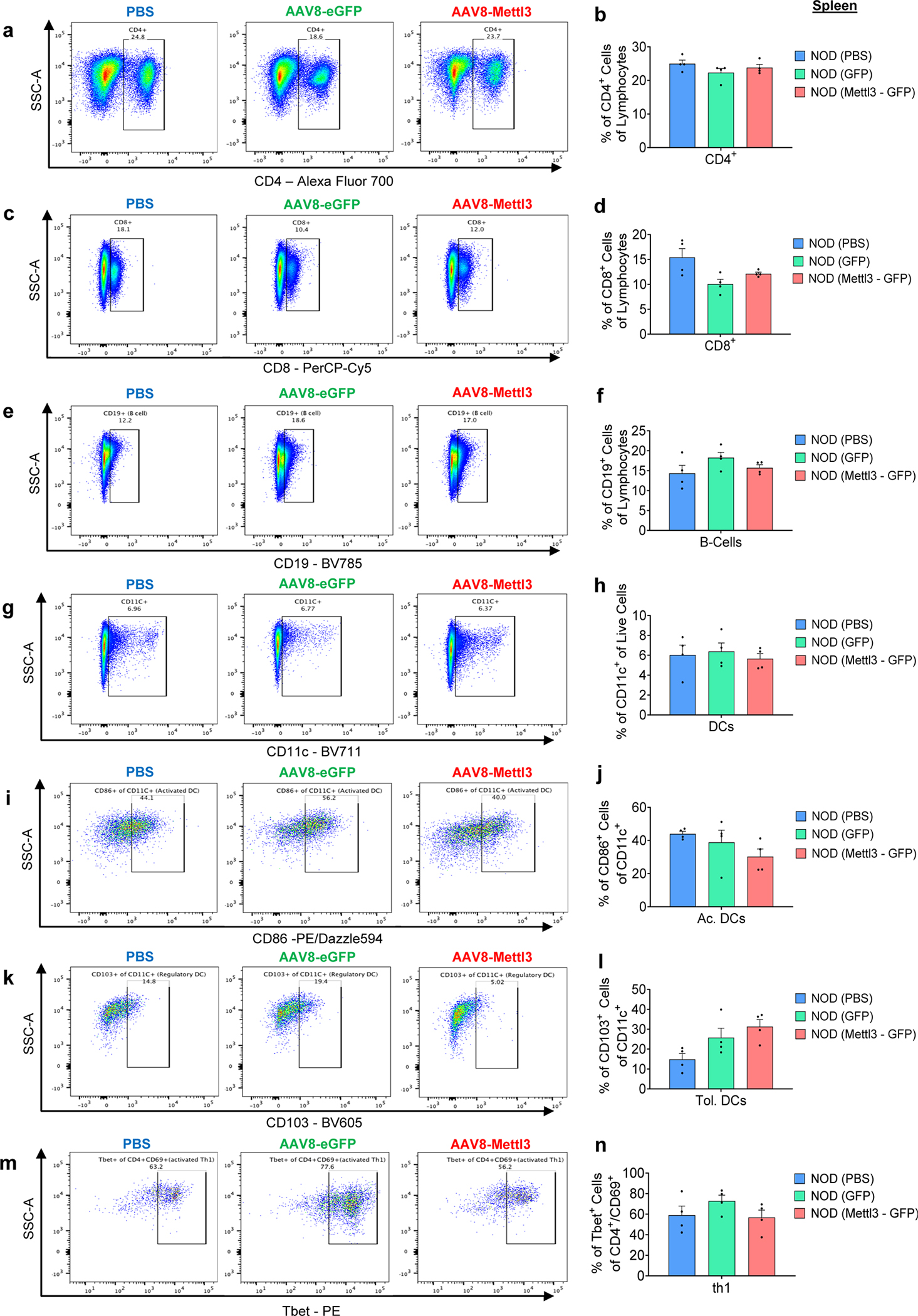
Immune cell profiling of NOD splenocytes (related to Fig. 7) **a-n,** Flow cytometry analyses of CD4 (a,b), CD8 (c,d), B-cells (e,f), dendritic cells (g,h), activated dendritic cells (i,j), tolerogenic dendritic cells (k,l), or th1 cells (m,n) isolated from spleens of NOD mice receiving PBS, eGFP, or Mettl3 overexpression (n=4/group). All samples in each panel are biologically independent. Data were expressed as means ± SEM. Statistical analysis was performed by One-Way ANOVA with Holm-Sidak test.

NOD mice present immune cell infiltration in salivary glands as well as pancreatic islets during progression of T1D^51^. All groups presented salivary gland infiltration (Fig. 7g), but only the AAV8-Mettl3 treated mice showed decreased immune infiltration in pancreatic islets (Fig. 7h) and consequently decreased insulitis scores compared to AAV8-eGFP or PBS-treated groups (Fig. 7i). Furthermore, an extensive immune cell profiling analysis of pancreatic lymph nodes (Extended Data Fig. 8a–n) revealed a decrease in the populations of CD4, CD8, and Th1 cells in AAV8-Mettl3-treated mice compared to AAV8-eGFP. Conversely, no notable changes in immune cell subpopulations were detected in splenocytes (see Extended Data Fig. 9). These findings were further corroborated by an increase in β-cell mass observed in AAV8-Mettl3-treated mice (Fig. 5j,k), along with elevated serum C-peptide levels compared to mice treated with AAV8-eGFP or PBS (Fig. 6l). Collectively, these results demonstrate that the prolonged upregulation of Mettl3 protects β-cells and effectively delays the onset of T1D in the NOD mouse model.

### Mettl3 overexpression hampers Oas immune response in NOD islets

To confirm the hypothesis that Mettl3 overexpression in mouse β-cells limits Oas upregulation in response to a T1D immune insult, we employed co-culture experiments (Fig. 7m). For this, we first transduced islets from 12-week-old female immunodeficient NOD SCID gamma (NSG) mice with AAV8-eGFP or AAV8-Mettl3, and then co-cultured them with either PBS or diabetogenic splenocytes from 12-week old diabetic NOD females (Fig. 7m).

Co-culture of islets from AAV8-eGFP-transduced NSG mice with diabetogenic splenocytes increased *Mettl3* expression compared to AAV8-eGFP islets treated with PBS (Fig. 7n). In addition, AAV8-Mettl3-islets presented a greater Mettl3 upregulation compared to AAV-eGFP-islets when also challenged with splenocytes (Fig. 7n). Mouse *Oas1* exhibits 8 paralog genes which have been described to differ in their antiviral activity^52^. Co-culture of diabetogenic splenocytes with islets transduced with AAV8-eGFP induced the upregulation of *Oas1a*, *Oas1c*, *Oas1g*, *Oas2*, and *Oas3* compared to islets transduced with AAV8-eGFP that were challenged with PBS (Fig. 7n). On the other hand, overexpression of Mettl3 (AAV8-Mettl3) in β-cells before co-culture with diabetogenic splenocytes blunted the upregulation of Oas genes compared to β-cells transduced with AAV8-eGFP and co-cultured with diabetogenic splenocytes (Fig. 7n). These data provide strong evidence for the existence of a conserved METTL3 regulation of the OAS innate immune response in both mouse and human β-cells.

### ROS-induced oxidative stress and endoplasmic reticulum stress constrain METTL3 upregulation

To examine the mechanisms involved in the dynamic regulation of METTL3, we considered recent reports describing the involvement of mitochondrial dysfunction, reactive oxygen species (ROS), and ER stress as contributors to the development of T1D ^53–55^.

**Fig. 8:**
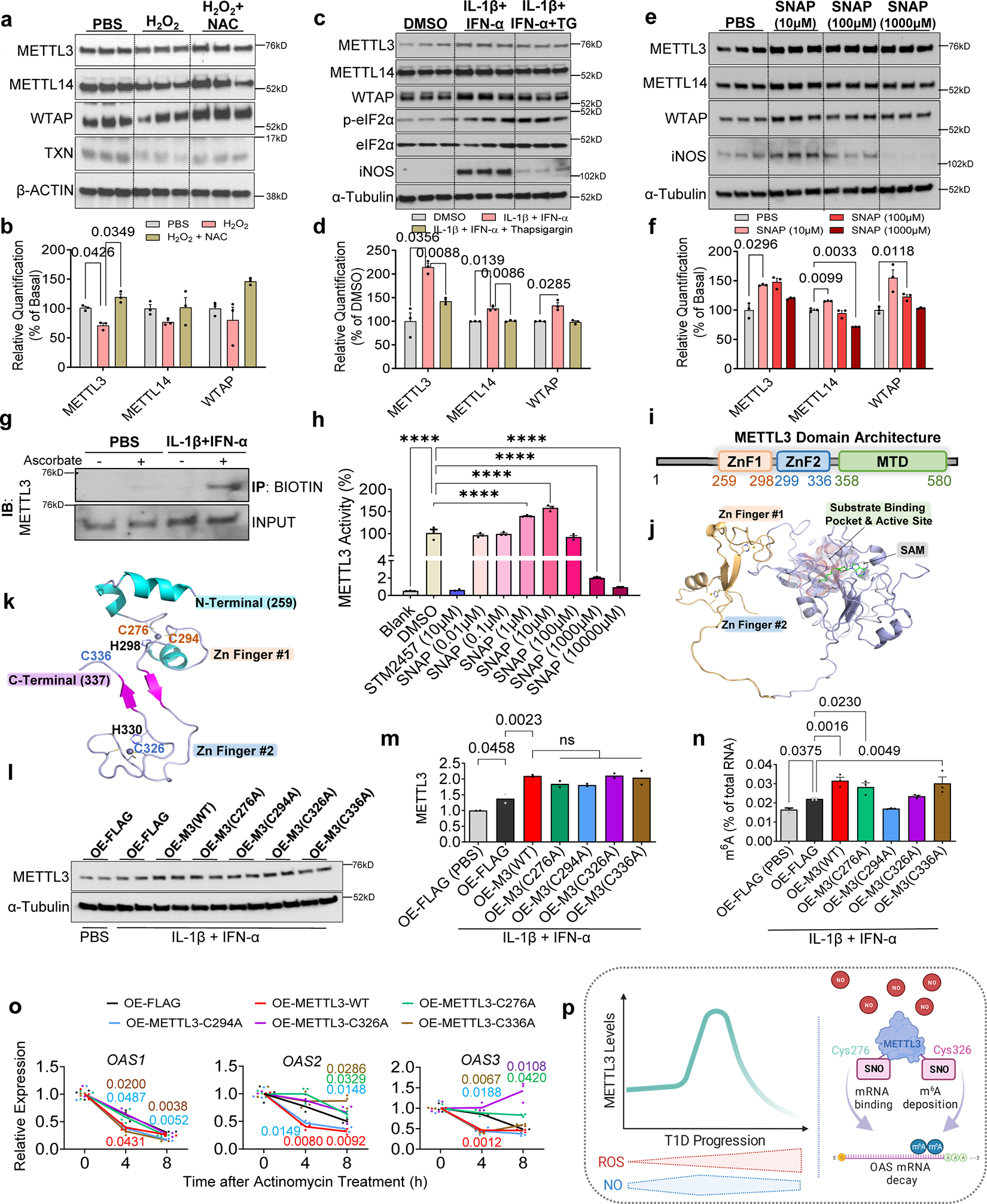
OAS mRNA stability is regulated by the S-nitrosylation of METTL3 in human β-cells. **a,** Western-blot analyses of indicated proteins in human islet cells treated with H_2_O_2_ or H_2_O_2_ plus N-acetyl cysteine (NAC) for 24h (n=3 independent biological samples/group). **b,** Protein quantification of indicated proteins related to (a). **c,** Western-blot analyses of indicated proteins in human islets treated with DMSO, IL1-β plus IFN-α pre-treated with thapsigargin plus IL1-β and IFN-α for a total of 24h (n=3 independent biological samples/group). **d,** Protein quantification of indicated proteins related to (c). **e,** Western-blot analyses of indicated proteins in human islets treated with PBS or represented doses of S-nitroso-N-acetyl-DL-penicillamine (SNAP) for 24h (n=3 independent biological samples/group). **f,** Protein quantification of indicated proteins related to (e). **g,** Western-blot analyses of biotin-switch assay on METTL3 in EndoC-βH1 cells treated with PBS or IL1-β plus IFN-α (n=3 independent experiments/group). **h,** METTL3:METTL14 complex methyltransferase activity with DMSO, STM2457 (a METTL3 inhibitor), or different concentrations of SNAP (n=3 independent experiments/group; n=2 independent experiments/STM2457). **i-j,** METTL3 protein domains representing zinc finger domains (ZnF) and methyltransferase domain (MTD). **k,** Structure of METTL3 zinc finger domains depicting the identified cysteines sensitive to S-nitrosylation. **l,** Western-blot analyses of METTL3 in EndoC-βH1 cells (n=2 independent experiments/group). **m,** Protein quantification of METTL3 related to (l). **n,** m^6^A levels measured by a colorimetric ELISA kit of total RNA isolated from EndoC-βH1 cells overexpressing the represented plasmids and treated with PBS or IL-1β plus IFN-α for 48h (n=3 independent experiments/group). **o,** qRT-PCR analyses of *OAS* genes after IL-1β plus IFN-α stimulation in EndoC-βH1overexpressing the represented plasmids after a time-course treatment with ActD (n=3 independent experiments/group). **p,** Model depicting the role of S-nitrosylation in controlling METTL3 function and OAS mRNA decay. All samples in each panel are biologically independent. Data were expressed as means ± SEM. Statistical analysis was performed by Two-Way ANOVA with Fisher’s LSD test or One-Way with ANOVA Holm-Sidak test in h and n. Source numerical data and unprocessed gel images are available in source data.

We began by challenging human islets with treatment of PBS, ROS (hydrogen peroxide alone), or H_2_O_2_ in combination with a ROS scavenger (N-acetyl cysteine- ‘NAC’). METTL3 protein levels were significantly downregulated by H_2_O_2_ and this downregulation was rescued in the presence of NAC (Fig. 8a,b).

**Extended Data Fig. 10:**
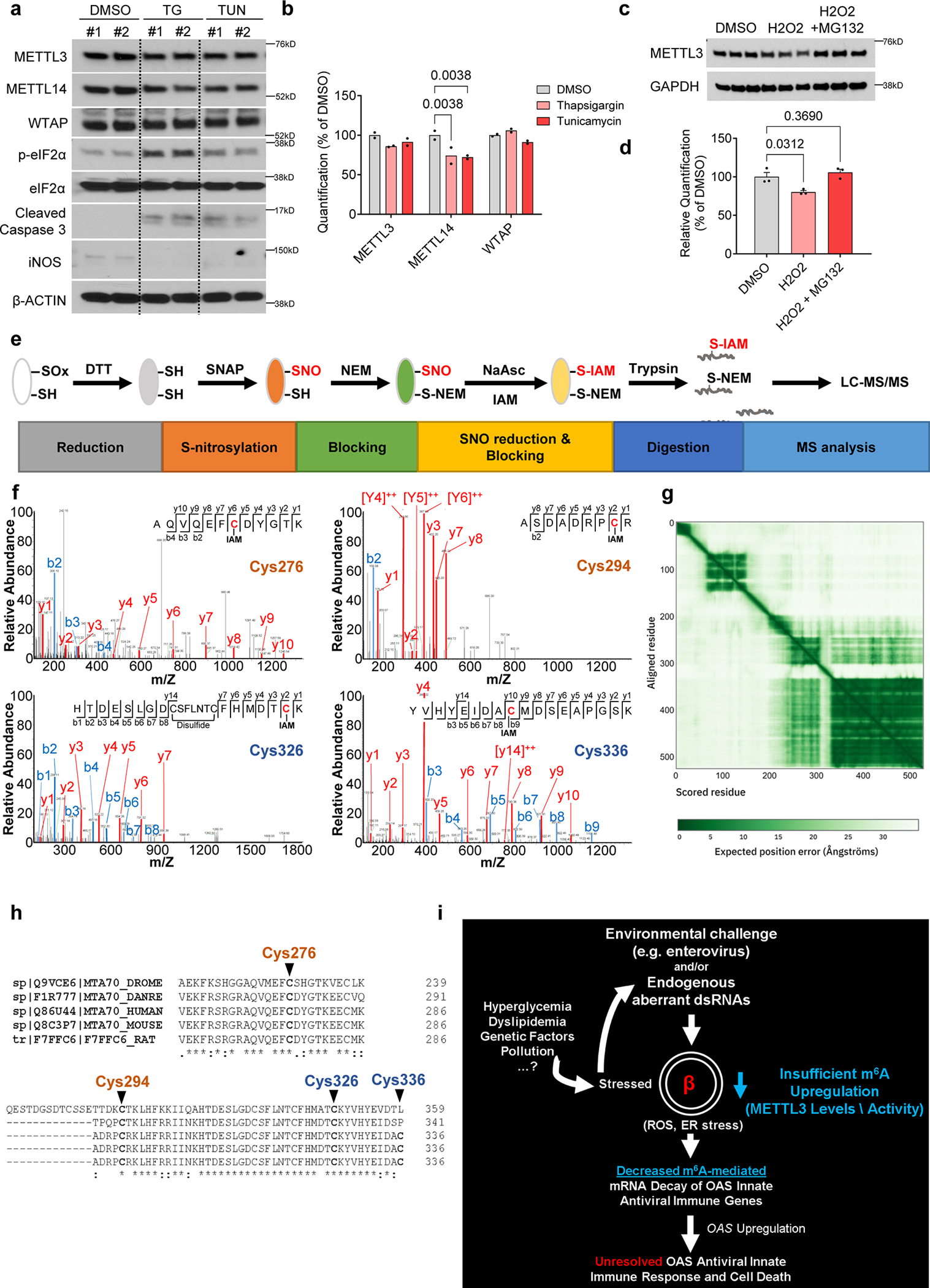
Summary schemes of experimental approaches and METTL3 protein structural modeling (related to Fig. 8) **a,** Western-blot analyses of indicated proteins in human islets cells treated with DMSO, 1μM thapsigargin, or 2μg/mL tunicamycin for 24h (n=2/group). **b,** Protein quantification of indicated proteins related to (a). **c,** Western-blot analyses of indicated proteins in EndoC-βH1 cells treated with DMSO, 25μM H_2_O_2_, or 25μM H_2_O_2_ + 5μM MG132 for 24h (n=3/group). **d,** Protein quantification of indicated proteins related to (c). **f,** MS/MS spectra of S-nitrosylated METTL3 peptides and the identification of cysteines C276, C294, C326, and C336 (n=2 replicates). **g,** Predicted aligned error related to Fig 8i–k. The color at position (x, y) indicates AlphaFold’s expected position error at residue x, when the predicted and true structures are aligned on residue y. **h**, CLUSTAL (1.2.4) multiple sequence alignment of METTL3 in represented species. **i,** Conceptual model of the role of METTL3 in controlling the OAS innate antiviral immune response by regulating the formation of deleterious dsRNAs and/or controlling viral response. The METTL3 response is decreased by the presence of ER stress and increased ROS levels, resulting from several potential factors such as hyperglycemia, dyslipidemia, genetic, or other environmental mediators such as pollution. Data were expressed as means ± SEM. Statistical analysis was performed by Two-Way ANOVA with Holm-Sidak test in b, and One-Way ANOVA with Holm-Sidak test in d.

We then examined the impact of ER stress on METTL3 regulation. First, we treated human islets with thapsigargin or tunicamycin (Extended Data Fig 10a). ER stress alone had a minor impact on m^6^A writers (Extended Data Fig 10b). To analyze the impact of ER stress in the context of T1D, we incubated human islets with thapsigargin before treatment with cytokines (Fig. 8c). Cytokine treatment led to the upregulation of all three components of the m^6^A writer complex (Fig. 8c,d). However, induction of ER stress by prior incubation with thapsigargin blunted upregulation of the m^6^A writers (Fig. 8c,d).

To study whether the downregulation of METTL3 was mediated by proteasomal degradation, we exposed EndoC-βH1 cells to H_2_O_2_, either alone or in conjunction with MG132 (Extended Data Fig 10c). As anticipated, H_2_O_2_ downregulated METTL3, a response that was effectively reversed with MG132 (Extended Data Fig 10d).

Next, we considered the mechanism(s) for the converse, i.e. METTL3 upregulation during the early stages of T1D. In this context, it is notable that while physiological levels of nitric oxide (NO) are protective, the persistent accumulation of ROS and NO to pathological levels may trigger β-cell apoptosis^53^. To examine the involvement of NO, we challenged human islets with different concentrations of an NO donor (S-Nitroso-N-acetyl-DL-penicillamine- ‘SNAP’). Physiological levels of SNAP increased METTL3 and iNOS protein abundance, while this response was lost in islets treated with high doses of SNAP (Fig. 8e,f). Altogether, these data support the concept that at early stages of T1D, an initial increase in NO induces upregulation of METTL3. However, as the disease progresses, the accumulation of NO and ROS in β-cells coupled with exacerbation of ER stress downregulates METTL3 and activates a persistent OAS response.

### Regulation of OAS mRNA decay in β-cells is dependent on S-nitrosylation of METTL3

To define the mechanism(s) involved in the regulation of METTL3 by NO, we first confirmed S-nitrosylation (SNO) of METTL3 in EndoC-βH1 cells (Fig. 8g). Next, we assessed METTL3 enzymatic activity. While low levels of NO increased METTL3 activity, this effect was reversed with high levels of NO (Fig. 8h). To identify the covalently modified Cys residues that are susceptible to SNO, we optimized a mass spectrometry-based protocol on human recombinant METTL3 protein treated with SNAP (Extended Data Fig. 10e). These analyses identified the SNO of four METTL3 Cys residues (Cys276, Cys294, Cys326, and Cys336) (Extended Data Fig. 10f). The domain architecture of human METTTL3 is characterized by the existence of two zinc finger domains (CCCH) and a methyltransferase domain (MTD)^21^ (Fig. 8i,j and Extended Data Fig. 10g). Interestingly, the METTL3 Cys residues sensitive to SNO were evolutionarily conserved (Extended Data Fig. 10h) and located in the zinc fingers (Fig. 8k).

We next tested the hypothesis that SNO of the identified cysteine residues were fundamental for the METTL3 regulation by performing site-directed mutagenesis and overexpressed either wild-type (WT) METTL3 constructs or mutant Cys METTL3 constructs in EndoC-βH1 cells. Cells overexpressing a FLAG empty plasmid (OE-FLAG) treated with cytokines presented upregulation of METTL3 compared to OE-FLAG cells treated with PBS (Fig. 8l,m). Overexpression of WT METTL3 in EndoC-βH1 cells was successful and importantly, all Cys mutants upregulated METTL3 similarly to WT METTL3 compared to a FLAG empty plasmid (Fig. 8l,m). However, while overexpression of WT METTL3 in EndoC-βH1 cells increased m^6^A levels in total RNA, this increase was blocked by mutating Cys294 and Cys326 (Fig. 8n). These data show that SNO at Cys294 and Cys326 residues in METTL3 is important for m^6^A deposition.

Finally, to determine which of the four cysteines directly influences METTL3 control OAS mRNA decay, we overexpressed FLAG, WT METTL3, or Cys mutant constructs independently in EndoC-βH1 cells. METTL3 overexpression accelerated the mRNA decay of *OAS1*, *OAS2*, and *OAS3* (Fig. 8o). Overall, mutant C294A overexpression cells behaved similarly to WT METTL3 (Fig. 8o), suggesting that SNO at this Cys residue does not control the mRNA decay of *OAS* despite impacting global m^6^A deposition. Overexpression of C336A accelerated the mRNA decay of *OAS1* and *OAS3* similarly to WT METTL3. However, SNO at this specific Cys was necessary for the mRNA decay of *OAS2* (Fig. 8o). On the other hand, overexpression of the mutants C276A and C336A behaved similarly to FLAG overexpressing cells and did not accelerate OAS mRNA decay as seen in the WT METTL3 overexpressing cells (Fig. 8o). Together, these data suggest that SNO regulates METTL3 and OAS mRNA decay by two different mechanisms: while SNO at Cys 326 impacts METTL3 global m^6^A deposition capacity, Cys 276 is likely important for the OAS RNA binding to METTL3 and does not disturb METTL3 m^6^A deposition in total RNA (Fig. 8p).

## DISCUSSION

There is growing evidence for a central role of the β-cell in triggering autoimmunity in T1D^56^. Here, we report that METTL3, is dynamically regulated and that m^6^A mRNA methylation provides key negative feedback on the antiviral innate immune response at the onset of T1D, preventing excessive and deleterious local inflammation. This protective effect is, however, dependent on the β-cell redox state and we describe SNO as a post-translational regulatory mechanism for METTL3 function and OAS mRNA decay.

We present several lines of evidence to validate the ability of METTL3 to control the mRNA decay of OAS genes via the m^6^A pathway. First, silencing *METTL3* increased OAS protein levels. Second, EndoC-βH1 cells deficient in METTL3 exhibited increased stability of OAS mRNA, while, overexpression of METTL3 accelerated OAS mRNA decay. Third, we demonstrated that the mRNA decay of OAS genes is mediated by the m^6^A readers YTHDF1 and YTHDF3. Finally, we report that the β-cell overexpression of Mettl3 in mice *ex vivo* dampened Oas upregulation in response to diabetogenic splenocytes. Although individual YTHDF proteins exhibited different behaviors^20^, it is worth mentioning that YTHDF2 directly participates in regulating mRNA decay^57^. Notably, we observed that YTHDF2 knockdown did not impact Oas mRNA decay in β-cells. This observation may be explained by the possibility that YTHDF2 is not a critical m^6^A reader in β-cells. Further work is necessary to examine the β-cell-specific role of YTHDF2.

In the context of the pathophysiology of T1D, repeated interactions with viruses may have resulted in the development of temporal safeguard mechanisms in host cells. For example, there may be development of activation thresholds for innate immune sensors before they can trigger elimination and/or prevent immuno-stimulatory endogenous nucleic acids. We report m^6^A as a safeguard mechanism to control the innate antiviral immune response thus establishing a link between antiviral innate immunity and T1D. Recent reports support this concept. For example, Gao and colleagues show that loss of Mettl3 in the fetal liver promotes the formation of deleterious dsRNAs and activation of innate immune pathways in the absence of a viral infection^26^. On the other hand, deletion of METTL3^24^ or METTL14^58^ in fibroblasts increases the mRNA stability of interferon β upon viral infection. Further studies are warranted to elucidate if m^6^A levels impact the β-cell susceptibility to viral infection and/or if downregulation of m^6^A levels in β-cells can trigger the formation of aberrant endogenous immune-stimulatory dsRNAs.

The m^6^A landscape of β-cells at T1D onset seemed to be distinct from that observed in established T1D. This argues for distinct features between the transcriptome and m^6^A landscape of β-cells at T1D onset – such as that observed in human islets and EndoC-βH1 cells treated with cytokines – compared to β-cells from patients with established T1D. This suggests that as T1D progresses, m^6^A decorates and controls genes important for β-cell function and identity. We have demonstrated in previous studies that silencing METTL3 or METTL14 in β-cells leads to cell-cycle arrest and apoptosis^29^. Therefore, it is reasonable to hypothesize that the downregulation of METTL3 during T1D progression accelerates the decline in β-cell mass, while its overexpression potentially limits this decline. Additional experiments are needed to show the direct link between METLL3 overexpression and β-cell mass preservation. We utilized input RNA-seq data to normalize m^6^A-seq in established T1D. Furthermore, we intersected the m^6^A-seq data in established T1D with a published resource on scRNA-seq in T1D β-cells to control for potential alterations in gene expression due to islet cell composition changes. Nevertheless, additional experiments are necessary to inform that changes in m^6^A are specific to β-cells in established T1D. Overall, these results point to m^6^A as a mechanism involved in the loss of β-cell identity and functional mass in the latter stages of T1D.

To begin to dissect the mechanism(s) involved in the dynamic regulation of METTL3 during T1D progression, we considered recent findings reporting increased accumulation of intracellular ROS prior to the development of T1D^53, 59^ and the upregulation of ER-stress markers in human T1D β-cells^60, 61^. We propose that during the early stages of T1D when the initial immune attack and cytokine release occurs, the rising levels of NO are within the physiological range and promote upregulation of METTL3. However, as the disease advances with progressive accumulation of NO and ROS, the heightened redox sensitivity of METTL3 triggers its downregulation and decreased enzymatic activity.

The upregulation of OAS genes at the onset of T1D despite an increase in METTL3 could be due to stressed β-cells failing to increase expression of the m^6^A writer in a timely manner to counterbalance the rapid rise of OAS. The fact that human islets pre-exposed to ER stress by thapsigargin treatment do not exhibit upregulation of METTL3 similar to control islets supports this contention. Furthermore, boosting Mettl3 overexpression in β-cells *in vitro* co-culture experiments was sufficient to limit the upregulation of Oas in islets from immunodeficient NSG mice in response to diabetogenic splenocytes.

FInally, other mechanisms could regulate the METTL3 m^6^A deposition independently of its total protein levels. For example, SUMOylation of METTL3 does not alter its stability, localization, or interaction with other m^6^A writers, but significantly represses its m^6^A methyltransferase activity^22^. SNO has been reported to be essential for diverse aspects of β-cell function^62^. We identified the four cysteines that are modified by SNO to be in the zinc finger domains of METTL3. This gains significance considering SNO of zinc fingers disrupts their structures and modulates enzymatic activity impacting the binding capacities of RNA binding proteins (RBPs)^63, 64^. A recent study has also reported that cysteines 294 and 326 in the zinc finger domains of METTL3 are essential for its enzymatic activity^65^. We observed that the SNO of the METTL3 Cys276 and Cys326 is needed for the mRNA decay of OAS in response to cytokines. Interestingly, while the mutation of Cys326 impacted m^6^A deposition, mutation of Cys276 did not alter m^6^A levels. This suggests that while SNO of Cys326 controls METTL3 enzymatic activity, SNO of Cys276 might regulate METTL3 structural binding to OAS mRNA. Overall, these results demonstrate that redox signaling controls METTL3 and OAS mRNA stability in β-cells at T1D onset.

In conclusion, we provide evidence that m^6^A methylation acts as a β-cell protective mechanism that controls the OAS innate immune response at the onset of T1D in mice and humans (Extended Data Fig. 10i). Our data suggests that increased m^6^A promotes accelerated mRNA decay of OAS genes in β-cells. Importantly, we observed that SNO represents a previously unidentified mechanism with the capacity to modulate METTL3 protein function and the potential mRNA binding affinity to OAS mRNA. Based on these results, we propose that therapeutic targeting of METTL3 before seroconversion or at T1D onset has the potential to promote β-cell survival and improve secretory function during disease progression.

## METHODS

### Study Approval

All animal experiments were conducted following the Association for Assessment and Accreditation of Laboratory Animal Care. All protocols were approved by the Institutional Animal Care and Use Committee of the Joslin Diabetes Center following NIH guidelines. All human studies and protocols used were approved by the Joslin Diabetes Center’s Committee on Human Studies (CHS#5–05). Formal consent from human islet donors was not required because samples were discarded islets from de-identified humans.

### Human islet isolation and processing

Human islets were obtained from the Integrated Islet Distribution Program (IIDP), Prodo Laboratories, ADI isletcore, and provided by Alvin C Powers MD (Vanderbilt U). Freshly isolated islets were cultured overnight (16h) in Miami Media #1A (Cellgro) upon arrival. Islets were then handpicked and seeded on ultra-low-attachment 6-well plates (Corning) (200 IEQ/well) for experiments. Snap-frozen control and T1D islets from ADI isletcore were immediately lysed in Trizol (ThermoFisher) upon arrival and stored at −80C for RNA isolation.

### EndoC-βH1 Cell Culture

The EndoC-βH1 cell line (EndoC-βH1^®^, Human Cell Design) was cultured and passaged as previously described^66^. Briefly, culture plates were coated with DMEM (glucose 4.5 g/L) (Gibco) containing PS (1%) (Gibco), fibronectin (2 μg/mL) (Sigma), and extracellular matrix (1% vol/vol) (Sigma) and incubated for at least 1 h in 5% CO2 at 37°C before the cells were seeded. EndoC-βH1 cells were grown on Matrigel/fibronectin-coated (Sigma) culture plates containing DMEM (glucose 1 g/L) (Gibco), BSA fraction V (2% wt/vol) (Roche Diagnostics), 2-mercaptoethanol (50 μM) (Gibco), nicotinamide (10 mM) (Sigma), transferrin (5.5 μg/mL) (Sigma), sodium selenite (6.7 ng/mL) (Sigma), and PS (1%) (Gibco)^66^.

### 266–6 Cell Culture

Briefly, 10cm culture plates were coated with DMEM (glucose 4.5 g/L) (Gibco) containing PS (1%) (Gibco), fibronectin (2 μg/mL) (Sigma), and extracellular matrix (1% vol/vol) (Sigma) and incubated for at least 1 h in 5% CO2 at 37°C before the cells were seeded. The 266–6 cells (CRL-2151, ATCC) were grown in 10cm culture plates containing DMEM (glucose 4.5 g/L) (Gibco), fetal bovine serum (10%) (Gibco), and PS (1%) (Gibco).

### PANC-1 Cell Culture

The PANC-1 cells (CRL-1469, ATCC) were grown in 10cm culture plates containing DMEM (glucose 4.5 g/L) (Gibco), fetal bovine serum (10%) (Gibco), and PS (1%) (Gibco).

### αTC-6 Cell Culture

The αTC-6 cells (CRL-2934, ATCC) were grown in 10cm culture plates containing DMEM (glucose 1 g/L) (Gibco), fetal bovine serum (15%) (Gibco), BSA fraction V (0.1% wt/vol) (Roche Diagnostics), non-essential amino acids (1%) (Gibco), and PS (1%) (Gibco).

### Human islets and cell treatments

#### Cytokines treatments:

Overnight cultured human islets or EndoC-βH1 cells were challenged with vehicle (PBS), IL-1β (50 U/ml; R&D Systems, USA), IFN-α (2000 U/ml; PBL Assay Science), IFN-γ (1000 U/ml; Peprotech), or a combination of IL-1β + IFN-α, or IL-1β + IFN-γ in respective culture media. After treatments, islets were then handpicked, washed twice with ice-cold DPBS (GIBCO) by self-sedimentation, and immediately lysed in Trizol for RNA isolation, RIPA buffer (ThermoFisher) for protein isolation, or fixed and embedded in agar for immunofluorescence staining as previously described^67^.

#### Thapsigargin treatments:

Overnight cultured human islets were treated with thapsigargin (1 μM; Selleckchem) or vehicle (DMSO) in Miami Media #1A for 16h and collected for protein isolation.

#### H_2_O_2_ and N-acetyl cysteine (NAC) treatments:

Overnight cultured human islets were treated with 25 μM of H_2_O_2_ (MiliporeSigma), or H_2_O_2_ plus 1 mM of NAC (Cayman chemical), or vehicle (PBS) in Miami Media #1A for 24h and collected for protein isolation.

#### S-Nitroso-N-Acetyl-D,L-Penicillamine (SNAP) treatments:

Overnight cultured human islets were treated with 10, 100, or 1000 nM of SNAP (Cayman chemical), or vehicle (DMSO) in Miami Media #1A for 24h and collected for protein isolation.

#### Actinomycin D treatments:

EndoC-βH1 cells were cultured as described above, and at 48h post-seeding/knock-down or overexpression were challenged with IL-1β + IFN-α as described above. At 72h post-seeding/knock-down or overexpression, cells were treated with 10μg/μL Actinomycin D (ThermoFisher) or DMSO for 0, 4, or 8h.

#### Cyclohexamide treatments:

EndoC-βH1 cells were cultured as described above, and at 48h post-seeding were challenged with IL-1β + IFN-α or PBS as described above for 24h. Cells were treated with 10μM of cyclohexamide (Cell Signaling) or DMSO for 4h after 20h of culture with cytokines and collected for protein isolation.

#### MG132 treatments:

EndoC-βH1 cells were cultured as described above, and at 48h post-seeding were challenged with IL-1β + IFN-α or PBS as described above for 24h. Cells were treated with 5μM of MG132 (Cell Signaling) or DMSO for 4h after 20h of culture with cytokines and collected for protein isolation.

### Transfections

#### Knock-down experiments:

Reverse transfections were performed as previously described ^29^. Briefly, EndoC-βH1 cells or dispersed human islet cells were mixed with Lipofectamine RNAiMAX Reagent (Life Technologies) and small interfering RNA complexes (Dharmacon) at a final concentration of 15 nmol/L siRNA according to manufacturer instructions. EndoC-βH1 cells were seeded at a density of 6×10^4^ cells/cm^2^ in Matrigel/fibronectin-coated (MiliporeSigma) culture plates. Human dispersed islets were seeded at a density of 5×10^4^ cells/cm^2^ on ultra-low attachment plates (ThermoFIsher) and allowed to form spontaneous pseudoislets. EndoC-βH1 cells and human pseudoislets were collected 96h post-transfection. ON-TARGETplus Non-Targeting Control Pool D-001810–10-05, ON-TARGETplus METTL3 siRNA L-005170–02-0005, ON-TARGETplus Human YTHDF1 siRNA L-018095–02-0005, ON-TARGETplus Human YTHDF2 siRNA L-021009–02-0005, ON-TARGETplus Human YTHDF3 siRNA L-017080–01-0005 (Dharmacon, USA).

#### Overexpression experiments:

EndoC-βH1 cells (1×10^6^ cells) were seeded in Matrigel/fibronectin-coated (MiliporeSigma) 6-well culture plates. After 24h, media was changed and cells were forward-transfected with 2μg of plasmid. c-FLAG pcDNA3 (addgene #20011) and pcDNA3/Flag-METTL3 (Addgene #53739) were obtained from Addgene. FLAG-tagged OAS1 (NM_016816.4), OAS2 (NM_002535.3), and OAS3 (NM_006187.4) plasmids were generated by VectorBuilder. Transfections were performed using Lipofectamine 3000 (Invitrogen) and Opti-MEM (Invitrogen) according to manufacturer protocols. Media was exchanged after 16h of transfection, and at 48h cells were further used for experiments including cytokine treatments and Actinomycin D/MG132 treatments.

### Mouse studies

Female NOD/shiLtJ (“NOD”; Jackson Laboratories #001976), NOR/LtJ (“NOR”; Jackson Laboratories #002050), and NOD.Cg-Prkdcscid Il2rgtm1Wjl/SzJ (“NOD NSG”; Jackson Laboratories #005557) mice were used. Mice were housed on a 12-h light/12-h dark cycle with water and food ad libitum. Mice were weaned and maintained on a chow diet (PicoLab^®^ mouse diet 20 – 5058). Female mice were used for all experiments throughout the study as male NOD mice do not develop T1D as consistently or within the same timeframe. Body weight and blood glucose were measured weekly for follow-up studies and mice were considered diabetic when two consecutive measurements of blood glucose exceeded 250 mg/dL. Serum C-peptide levels were measured using ELISA kits (Crystal Chem) according to manufacturer guidelines. All mice were kept in a specific pathogen-free facility in the Animal Facility at Joslin Diabetes Center, and animal protocols were approved by the Institutional Animal Care and Use Committee (IACUC). Sample sizes for animal experiments were chosen based on experience in previous in-house studies of metabolic phenotypes and to balance the ability to detect significant differences with minimization of the number of animals used following NIH guidelines.

#### Mouse Islet Isolations:

Islets were isolated from female NSG mice as previously described ^68^. In brief, 3-month-old mice were anesthetized and their pancreas was infused with liberase (Roche). Following incubation at 37°C for 17 min the digested pancreases were washed, filtered through a 400μm filter, and run on a Histopaque (Sigma, USA) gradient. The purified islets were handpicked, counted, and cultured overnight in 7mM glucose RPMI media (Gibco, USA) containing 10% FBS and 1% PS) (Gibco, USA).

#### β-cell sorting by FACS:

Overnight cultured mouse islets were dispersed with a solution of 1 mg/ml trypsin and 30 μg/ml DNase followed by incubation for 15 min at 37°C. During the digestion, the islets were vortexed every 5 min for 10s. Cold media including serum was added to stop the digestion, and the cells were washed two times in DPBS containing 1% fatty-acid-free bovine serum albumin (BSA). Before sorting islet cells were filtered through a 35 μm filter and sorted using MoFlo Cytometer (Dako), where cells were gated according to forward scatter and then sorted based on endogenous fluorescence ^32^ and CD45 staining (Biolegend # QA17A26).

#### Co-culture of splenocytes and islets:

Total splenocytes were purified as previously described ^36^. Briefly, freshly harvested spleens of 12–13 weeks-old female NOD mice with early diabetes were filtered through a nylon mesh by followed lysis of the red blood cells with ACK Lysing buffer (Lonza). After starvation, 100 size-matched islets from NOD NSG mice were co-cultured with NOD splenocytes in 5 mmol/L glucose RPMI at a ratio of 1:10 as previously described ^69^. At 48h islets were hand-picked, washed in ice-cold DPBS, and lysed in Trizol for RNA isolation.

#### NODs immune cell profiling by FACS:

Splenocytes and pancreatic lymph nodes were harvested and stained with a viability dye Fvd (APC-Cy7, #6508614, ebioscience). Subsequently, various antibodies were applied at a 1:300 dilution, as follows: CD4 (Alexa Fluor-700, #2081383, ebioscience), CD8a (PerCP-Cy5.5, #2151510, ebioscience), CD25 (Pacific Blue, #102022, Biolegend), CD69 (PE-Cy5, #104510, Biolegend), CCR6 (PE-Cy7, #129816, Biolegend), CD19 (BV785, #115543, Biolegend), CD11C (BV711, #117349, Biolegend), CD27 (BV650, #124233, Biolegend), CD103 (BV605, #121433, Biolegend), CD86 (PE/Dazzle594, #105042, Biolegend), RorT (APC, #2193857, ebioscience), FOXP3 (FITC, #2290357, ebioscience), Tbet (PE, #644810, Biolegend). Isotype controls were employed to mitigate the effects of nonspecific binding and to ensure proper gating. All incubations were conducted on ice and protected from light. A minimum of 100,000 cells were counted using a Fortessa flow cytometer (BD Biosciences) and subsequently analyzed using FlowJo software.

#### *In vivo* Mettl3 overexpression:

Adeno-associated virus serotype 8 (AAV8) overexpressing Mettl3 (NM_019721.2) or eGFP under the control of rat insulin II promoter (addgene # 15029) with a WPRE element were synthesized by VectorBuilder. Briefly, 4-week-old NOD female mice received an intraperitoneal injection of 200 μl PBS + 0.01% Pluronic F-68 (Sigma) containing 1×10^11^ gene copies (gc)/mouse of AAV8 overexpressing eGFP or Mettl3 and were followed for 20 weeks.

### RNA isolation and RT-PCRs

Total RNA was isolated as previously described ^70^. In brief, high-quality total RNA (>200nt) was extracted using standard Trizol reagent (Invitrogen) according to manufacturer instructions and the resultant aqueous phase was mixed (1:1) with 70% RNA-free ethanol and added to Qiagen RNeasy mini kit columns (Qiagen) and the kit protocol was followed. RNA quality and quantity were analyzed using Nanodrop 1000 and used for reverse transcription using the high-capacity cDNA synthesis kit (Applied Biosciences). cDNA was analyzed using the ABI 7900HT system (Applied Biosciences) and gene expression was calculated using the ^ΔΔ^Ct method. Data were normalized to GADPH.

### m^6^A-IP-qPCR

EndoC-βH1 cells were grown as described above in 15cm matrigel/fibronectin-coated plates and treated with IL-1β + IFN-α or PBS for 48h. Total RNA was isolated as described above and 50μg were fractionated using NEBNext Magnesium RNA Fragmentation Module (NEB, # E6150S). 18μl of total RNA were used for m^6^A-immunoprecipitation (m^6^A-IP) using the EpiMark N6-Methyladenosine enrichment kit (NEB, #E1610S) according to the kit protocol and 2μl kept as input. The resulting IP and input RNA were cleaned up using the Monarch RNA Cleanup Kit (NEB #T2030) and 200ng was used for reverse transcription using the high-capacity cDNA synthesis kit (Applied Biosciences). cDNA was analyzed using the ABI 7900HT system (Applied Biosciences) and gene expression was calculated using the ^ΔΔ^Ct method.

### Protein isolation and Western blotting

Total proteins were harvested from cell lines using M-PER (Thermo Fisher), and tissue (e.g. islets) using RIPA protein extraction reagent (Thermo Fisher) supplemented with proteinase and phosphatase inhibitors (Sigma) according to standard protocol. Protein concentrations were determined using the BCA standard protocol followed by the standard western immunoblotting protocol of proteins. Primary antibodies used for western-blotting with a 1:1000 dilution included METTL3 (#195352, Abcam), METTL14 (#HPA038002, Sigma), WTAP (#60188–1-Ig, Proteintech), OAS1 (#14955–1-AP, Proteintech), OAS2 (#19279–1-AP, Proteintech), OAS3 (#21915–1-AP, Proteintech), RNASEL (#22577–1-AP, Proteintech), ADAR1 (#14175, Cell Signaling), phospho-STAT1 (#9167, Cell Signaling), STAT3 (#12640, Cell Signaling), iNOS (#ab178945, Abcam), phospho-eIF2a (#3398, Cell Signaling), eIF2a (#5324, Cell Signaling), Thioredoxin 1 (#2429, Cell Signaling), β-ACTIN (#4970, Cell Signaling), αTubulin (#7291, Abcam), GAPDH (#5174, Cell Signaling). The blots were developed using chemiluminescent substrate ECL (ThermoFisher) and quantified using Image studio Lite Ver. 5.2 software (LICOR).

### Pancreas immunostaining and analyses

Mouse pancreas was collected and fixed in 4% formaldehyde at 4°C overnight, followed by paraffin embedding. Five-micron-thick slides were cut and subjected to immunostaining. Slides were heated in 10mM sodium citrate, followed by blocking with donkey serum, and incubated with various primary antibodies: Proinsulin (DSHB, #GS-9A8, dilution 1:2500), Insulin (Abcam, #ab7842, dilution 1:500), Glucagon (Sigma, #G2654, dilution 1:8000), Somatostatin (Abcam, #ab30788, dilution 1:10000), METTL3 (Abcam, #195352, dilution 1:5000). Specific signals were detected by using fluorescence-conjugated secondary antibodies (Jackson Immunoresearch, Alexa 488, Alexa 594, and AMCA). Images were captured using Zeiss AXIO Imager A2 upright fluorescence microscope. Insulitis was evaluated as reported previously ^36^. Quantification of β-cell mass was performed as previously described ^67^.

### Measuring total m^6^A levels

Total m^6^A levels were measured by employing LC-MS/MS or a quantitative colorimetric ELISA.

#### LC-MS/MS quantification of m^6^A:

Total m^6^A levels among all adenosines were measured by triple-quad LC-MS/MS. We first purified mRNA from human isleťs total RNA by two rounds of polyA selection using polyA beads. 50 ng of purified mRNA were subject to digestion by 1 Unit of nuclease P1 (Sigma #N8630–1VL) in 25 μL of buffer containing 20 mM of NH4Ac at 42 degrees for 2 hours followed by phosphatase treatment using 1 μL of FastAP Thermosensitive Alkaline Phosphatase (ThermoFisher #EF0651) at 37 degrees for 1 hour. The digested nucleotides were filtered by a 0.22 μm syringe filter (Millipore) and then analyzed by a C18 reverse phase column on HPLC (Agilent) followed by triple quad MS/MS quantification (Sciex). The concentration of each type of nucleotide was calibrated by standard curves measured from pure nucleoside standards in each experiment. The m^6^A/A ratio was computed using the estimated m^6^A and A-concentrations.

#### Colorimetric quantification of m^6^A:

EpiQuik m^6^A RNA Methylation Quantification Kit (EpigenTek) was used to measure the percentage of m^6^A methylation level in total RNA. EndoC-βH1 cells harboring WT or mutant METTL3 overexpression were used according to the protocols of the manufacturer using the kit provided negative control, and positive control, and our samples consisting of 200μg of total RNA from EndoC-βH1 cells. The m^6^A percentage in total RNA was calculated using the following formula: m^6^A% = (Sample OD − NC OD) ÷ S)/(PC OD − NC OD) ÷ p) × 100%. NC: negative control; PC: positive control; S: the amount of input sample RNA; p: the amount of input positive control. Equal amounts of RNA samples were used.

### m^6^A immunoprecipitation and sequencing

For patient islet samples, polyA-selected mRNA was adjusted to 15 ng/μL in 100ul and fragmented using a Bioruptor ultrasonicator (Diagenode) with 30s on/off for 30 cycles. m^6^A-immunoprecipitation (m^6^A-IP) was performed using the monoclonal m^6^A antibody from the EpiMark N6-Methyladenosine enrichment kit (NEB, #E1610S). Input and eluted total RNA from m^6^A-IP were used to prepare libraries with Takara Pico-Input Strand-Specific Total RNA-seq for Illumina v2 (Takara). Sequencing was performed on Illumina Nova-seq according to the manufacturer’s instructions. Approximately 30 million paired-end 150-bp reads were generated for each sample.

### Differential methylation analysis for m^6^A sequencing

Human genome sequences and gene annotations were downloaded from the UCSC golden path version hg38. We generated genome indexes using the genomeGenerate module of the STAR aligner ^71^ with sjdbOverhang as 52 for 53-bp reads and 59 for 60-bp reads. The reads were trimmed for adapters and poly(A/T) tails, and then filtered by sequencing Phred quality (>= Q15) using fastp ^72^. We aligned the adapter-trimmed reads to the genome using STAR with the two-pass option and indexed the BAM files with samtools ^73^. Using the R package RADAR ^74^, we counted the mapped reads in 50-bp consecutive bins of each gene for each pair of input and m^6^A immunoprecipitation (IP) samples. Counts were normalized for library size and IP counts were adjusted for expression level by the gene-level read counts of input libraries. Bins with average IP-adjusted counts lower than 10 in both CTRL and CASE groups were removed. Then bins that were not enriched in IP were also filtered out. To construct PCA plots, we used the removeBatchEffect function in the limma package ^75^ to remove the subject effect for the human islets; the clone effect for the EndoC-βH1 cells; the batch, gender, age, and BMI effects for the T1D islet study. We performed differential methylation analysis of count data using the R package DESeq2 ^76^. To consider the pairing of m^6^A IP and input, we used the normalized, expression-level-(i.e. input)-adjusted, and low-read-count-filtered IP counts. Using the Wald test, we tested for significant effects of cytokine treatment or T1D on the m^6^A enrichment/depletion. To discover cytokine treatment effects in human islets or EndoC-βH1 cells, we performed paired tests so that each cytokine-treated sample was compared to its own paired baseline sample. To discover T1D effects in human islets using all controls, we adjusted for batch, gender, age, and BMI. We then merged the neighboring significant bins. P-values of these bins were combined by Fisher’s method ^77^. We adjusted for multiple testing using the Benjamini-Hochberg false discovery rate (FDR) controlling procedure.

### Differential expression analysis of m^6^A-sequencing input samples

The input libraries of m^6^A sequencing are essentially mRNA sequencing libraries, so we performed gene-level differential expression analysis on them. After STAR alignment, alignments were assigned to genomic features (e.g. the exons for spliced RNAs) using featureCounts ^78^. Multi-mapping reads were counted as fractions. R package DESeq2 ^76^ was used to test for differential expression where sequencing batch, gender, and age were included as covariates. We adjusted for multiple testing using the Benjamini-Hochberg FDR procedure.

### RNA-seq analyses of sorted NOD mouse β-cells

Reversely stranded 100 bp single-end reads were trimmed for adapters and filtered by sequencing Phred quality (>= Q15) using fastp^72^. Reads were aligned to the mouse transcriptome (Ensembl version 98) using kallisto^79^ and transcript counts were converted to gene counts using tximport^80^. To filter out low-expressing genes, we only kept genes that had counts per million (CPM) more than 1 in at least 3 samples. We then normalized counts by weighted trimmed mean of M-values (TMM)^81^. To use linear models in the following analysis, we transformed counts into logCPM with Voom^82^. To discover the differential genes, we used the linear regression modeling R package limma^75^, which applied moderated t-tests to detect genes that are differentially expressed between groups. We adjusted for multiple testing using the Benjamini-Hochberg FDR procedure.

### RNA-seq of EndoC-βH1 cells overexpressing OAS

Sequencing was performed by BGI. Briefly, RNA samples were denatured at an appropriate temperature to disrupt their secondary structure, and mRNA was enriched using magnetic beads attached with oligo (dT). A reaction system was configured, and RNAs were fragmented after a set duration at an optimal temperature. The first-strand synthesis reaction system was added to the fragmented mRNA to synthesize the first-strand cDNA. Next, the second-strand synthesis reaction system (including dUTP) was prepared, and the second-strand cDNA was synthesized. Following the configuration and setup of reaction systems and programs, double-stranded cDNA fragments underwent end repair, and a single 'A' nucleotide was added to the 3' ends of the blunt fragments. Adaptors were then ligated to the cDNAs, with corresponding reaction systems and programs. PCR reactions were set up to amplify the cDNAs. Library quality control protocols were chosen based on specific product requirements. Denaturation resulted in the production of single-stranded PCR products. The reaction system and program for circularization were subsequently configured and set up. This process generated single-stranded circularized products, while linear DNA molecules that did not cyclize were digested. Single-stranded circular DNA molecules were replicated through rolling cycle amplification, producing DNA nanoballs (DNBs) containing multiple copies of DNA. High-quality DNBs were loaded onto patterned nanoarrays using high-intensity DNA nanochip techniques and sequenced using the combinatorial Probe-Anchor Synthesis (cPAS) method. RNA-seq analyses were performed as described above for NOD β-cells, with the difference that to filter out low expressing genes, we kept genes that have counts per million (CPM) more than 0.49 in at least 4 samples.

### Re-analyses of single-cell RNA-seq dataset

Data was obtained from GEO under the accession number GSE121863^33^. First, we computed some quality control metrics with R package scater^83^. We removed outlier cells whose library size and number of expressed genes were too low or whose proportion of counts assigned to mitochondrial genes was too high using thresholds of 2000, 1000, and 10%. We removed genes that have average counts of 0. We clustered similar cells together using a graph-based clustering algorithm and using genes that have average counts of more than 0.1. The algorithm normalizes the cells in each cluster using the deconvolution method^84^. Finally, it performs scaling to ensure that size factors of cells in different clusters are comparable. Next, we estimated the technical noise by assuming the noise follows a Poisson distribution. We used the 1,000 genes that have the largest biological variations and performed Principal Component Analysis (PCA). We selected the first 100 PCs for the following analysis. We constructed tSNE plots from the PCs where each point represented a cell and was colored according to the variable diabetes. The insulin expression levels had 4 modes. We then used the Gaussian finite mixture model to identify cells in the 3rd and 4th mode, i.e. those with the highest insulin expression^85^. To perform differential gene expression in β-cells, we selected β-cells and analyzed genes that are expressed in at least 10 cells using limma^75^, which applied moderated t-test to detect genes that are differentially expressed between the established T1D and Non-T1D. We adjusted for multiple testing using the Benjamini-Hochberg FDR procedure.

### Pathway enrichment analysis

Pathway enrichment analyses were performed using ConsensusPathDB^86^ or Metascape^87^ using default settings. GO terms tree were constructed using Cytoscape^88^. Protein-protein functional networks were constructed using STRING using default settings^89^.

### Biotin Switch assay

We used the biotin switch method that converts –SNO into biotinylated groups using a detection kit (Cayman Chemical), to detect S-nitrosylation of METTL3 in EndoC-βH1 cells treated with IFN-α and IL-1β according to the protocols of the manufacturer.

### LC-MS/MS analysis on METTL3 S-nitrosylation (SNO)

10 μg recombinant human METTL3 was incubated with 1 mM DTT for 30 min at room temperature (RT), and buffer was exchanged into 30 μL of 50 mM HEPES (pH 7.4) containing 1 mM EDTA, 0.1 mM neocuproine and 0.05% SDS using 30k spin columns. METTL3 was incubated with and without 200 μM SNAP in dark at RT for 1 hr. Excessive SNAP was removed by buffer exchange with 50 mM HEPES (pH 7.4). Free thiols in all samples were blocked by 20 mM NEM at RT for 30 min. NEM was removed by washing with 50 mM NH_4_HCO_3_(pH 8) containing 1 mM EDTA, 0.1 mM neocuproine, and 8M urea. SNO modifications in proteins were reduced with 20 mM sodium ascorbate and alkylated by 20 mM iodoacetamide at RT for 1 hr. Proteins were then digested by trypsin (enzyme to protein ratio = 1: 20) overnight. Peptides were eluted by centrifugation in 50 mM NH_4_HCO_,_ and the concentration of each sample was adjusted to 0.05 μg/μL for LC-MS/MS. LC-MS/MS analysis was conducted using a nanoAcquity UPLC system (Waters) coupled to a Q-Exactive Mass Spectrometer as previously described ^90^. MS/MS raw data were searched against the Uniprot FASTA file of Homo Sapiens using MS-GF+ algorithm. Dynamic modifications included the oxidation of methionine (15.9949 Da), NEM on cysteine (125.0477 Da), and iodoacetamide on cysteine (57.0215 Da).

### Site-directed mutagenesis

Site-directed mutagenesis was performed by Genscript. Briefly, vector pcDNA3/Flag-METTL3 (Addgene #53739) was transformed and the plasmid was extracted with Axygen kit (Corning). Next, sequence verification and enzyme digestion were performed for vector verification. Plasmid was linearized by digestion with HindIII and XboI to obtain a ~5.2 kb vector backbone. Gene fragments were prepared by using two specific pairs of primers with overlap for each construct. Gene fragment amplification was performed by two rounds of PCR to generate the target gene fragment with the designed point mutation using the WT as a template. The PCR product was purified and cloned, and ligated with a linearized vector with T4 ligase. Gene products were transformed into competent cells and single colonies were picked for screening and sequence verification.

### METTL3 protein structure modelling

Structural figures of METTL3 were generated using Pymol (Delano) and coordinates from AlphaFold accession number AF-Q86U44-F1-model_v4 highlighting the zinc finger cluster and catalytic domain inter-domain interactions (also shown in the predicted aligned error (PAE) grid in Extended Data Figure 10g) ^91^. The zinc finger cluster is shown in light-orange and the catalytic SET domain in light-blue ribbon format and the SAM cofactor in stick format. The majority of the active site, including the cofactor and substrate binding pockets, is shown in dotted surface format. The solution structure of the zinc finger cluster from PDB 5YZ9 ^65^ is also shown in greater detail in ribbon format including the zinc atoms and coordinating cysteine residues implicated in S-nitrosylation.

### METTL3/METTL14 methyltransferase activity

METTL3/METTL14 activity was analyzed using the METTL3/METTL14 Complex Chemiluminescent Assay Kit (BPS Biosciences, #79614) in the presence of DMSO, SNAP (Cayman chemical), or STM2457 (Selleckchem) according to the kit protocol.

### Statistics and Reproducibility

The sample size was chosen based on having an 80% power in detecting a difference at a significant level of α=0.05. Sample sizes for animal experiments were chosen based on experience in previous in-house studies of metabolic phenotypes and to balance the ability to detect significant differences with minimization of the number of animals used following NIH guidelines. Replication attempts were successful. All replication experiments were included in the study. All experiments were performed using a minimum of 3 biological replicates unless specified in the legends. All cell line experiments were performed in 3 independent experiments. Animals were randomly assigned to experimental groups and matched for age and gender. β-cell mass analyses and insulitis scores were performed blindly. In-vitro experiments with genetic manipulation such as knock-down experiments were performed with awareness of groups to distinguish scramble from knock-down/overexpressing cells. Blinding was not relevant or possible in other experiments.

## Extended Data

## Supplementary Material

Supplementary Table 1

## Data Availability

m^6^A-sequencing and RNA-sequencing data in human islets and EndoC-βH1 cells have been deposited with the National Center for Biotechnology Information Gene Expression Omnibus under accession code GSE228267. RNA-sequencing in NOD mouse FACS-sorted β-and non-β-cells have been deposited under the accession code GSE228219. RNA-sequencing data in EndoC-βH1 cells harboring OAS overexpression have been deposited under the accession code GSE244273. Mass spectrometry data on METTL3 S-nitrosylation have been deposited with Mass Spectrometry Interactive Visual Environment (MassIVE) under the accession code MSV000091547. Source data have been provided in Source Data. All other data supporting the findings of this study are available from the corresponding author upon reasonable request.
